# Optimizing phage therapy for carbapenem-resistant *Enterobacter cloacae* bacteremia: insights into dose and timing

**DOI:** 10.1128/aac.01683-24

**Published:** 2025-02-26

**Authors:** Shi-Yong Fu, Xiu-Zhen Chen, Peng-Cheng Yi, Jie Gao, Wei-Xiao Wang, Shuang-Lin Gu, Jing-Han Gao, Du-Xian Liu, Han-Feng Xu, Yi Zeng, Chun-Mei Hu, Qin Zheng, Wei Chen

**Affiliations:** 1 Department of Oncology, the Second Hospital of Nanjing, Affiliated Hospital to Nanjing University of Chinese Medicine 531909 , Nanjing, China; 2 Department of Tuberculosis, the Second Hospital of Nanjing, Affiliated Hospital to Nanjing University of Chinese Medicine 531909 , Nanjing, China; 3 Clinical Research Center, the Second Hospital of Nanjing, Affiliated Hospital to Nanjing University of Chinese Medicine 531909 , Nanjing, China; 4 Department of Pathology, the Second Hospital of Nanjing, Affiliated Hospital to Nanjing University of Chinese Medicine 531909 , Nanjing, China; 5 The Clinical Infectious Disease Center of Nanjing, Nanjing, China; Johns Hopkins University School of Medicine, Baltimore, Maryland, USA

**Keywords:** *Enterobacter cloacae *complex, OmpA, receptor, host range, phage therapy, dose optimization, prophylactic use

## Abstract

The increase in multidrug-resistant (MDR) *Enterobacter cloacae* complex (ECC) infections, particularly those resistant to carbapenems, underscores the urgent need for alternative therapies. Phage therapy, with its specific bactericidal action, offers a promising solution. However, there remains a shortage of well-characterized ECC-targeting phages, and dosing and timing optimization for ECC-specific phage cocktails is largely unexplored. In this study, we isolated and characterized three novel lytic phages with diverse genome sizes and host ranges. Notably, ФEBU8 demonstrated broad-spectrum activity, lysing both *Enterobacter* species and *Acinetobacter baumannii*. ФECL22 displayed stability across a wide temperature range (4–50°C), pH tolerance (6–10), and a burst size of 19 PFU/cell, with OmpA identified as its receptor. Our formulated phage cocktail, comprising ФEBU8, ФECL22, and ФECL30, effectively rescued mice with *E. cloacae* bacteremia in a dose-dependent manner, with a mid-dose regimen showing particularly strong efficacy. Immediate phage administration achieved full survival, whereas a combined prophylactic and therapeutic regimen (“−24 + 6”) also resulted in 100% survival. These findings highlight the critical roles of dosing and timing in optimizing phage therapy for carbapenem-resistant *Enterobacter* infections, with prophylactic use providing a valuable window for delayed treatment and a promising strategy for combating severe bacterial infections.

## INTRODUCTION

The increase in antimicrobial resistance (AMR) among *Enterobacteriaceae*, particularly the increasing prevalence of carbapenem-resistant strains, presents an urgent global health challenge. These multidrug-resistant organisms are responsible for a high burden of morbidity and mortality, particularly in hospital-acquired infections, where they significantly complicate patient management and outcomes. Among the *Enterobacteriaceae*, the *Enterobacter cloacae* complex (ECC) is especially concerning, as it has been identified as a major pathogen in bloodstream, respiratory, and urinary tract infections, particularly in immunocompromised patients (1, 2). Furthermore, ECC possesses a remarkable ability to acquire resistance genes through horizontal gene transfer and intrinsic resistance to multiple classes of antibiotics (3). These organisms belong to the "ESKAPEE" group, a group of pathogens prioritized by the World Health Organization (WHO) for their propensity to develop resistance and cause severe infections. The 2023 annual report of China Antimicrobial Resistance Surveillance System (CARSS) showed that the ECC has exhibited alarming rates of carbapenem resistance, up to 9.2% in China (4). The rising prevalence of carbapenem-resistant ECC (CRECC) further limits therapeutic options and a driving interest in alternative therapies (5–7).

Polymyxin B remains the last-resort treatment for CRECC infections. However, the emergence of polymyxin-resistant strains has undermined this last line of defense (8). Phage therapy has re-emerged as a promising approach to combat multidrug-resistant bacterial infections due to its unique mode of action, which can specifically target and lyse bacteria without disrupting the host microbiome (9, 10). Unlike conventional antibiotics, bacteriophages can self-replicate at the infection site, amplifying in response to bacterial density, which presents an advantage in reducing bacterial burden *in situ*. However, despite these advantages, phage therapy also presents several challenges. One concern is the potential for phage resistance, where bacteria may evolve mechanisms to evade phage-mediated lysis, thereby reducing therapeutic efficacy (11). Additionally, the regulatory hurdles associated with phage therapy, such as the lack of standardized protocols for phage isolation, formulation, and clinical testing, complicate its widespread adoption (12). Moreover, phage therapy’s efficacy can be influenced by the availability of suitable phages for specific pathogens, as well as the need for personalized treatment regimens (13). The success of phage therapy also hinges on factors such as dose and timing of administration. Several studies have shown that administering a phage cocktail at the right dose and within an optimal therapeutic window can improve survival rates and clinical outcomes in infection models (14). Although considerable research has focused on phage therapy against common multidrug-resistant (MDR) pathogens, *Enterobacter*-specific phage therapies remain underexplored, in part due to a relative shortage of characterized phages for ECC. To date, no studies have been conducted to optimize dosing and timing specifically for phage cocktails targeting ECC.

In this study, we investigated the efficacy of a newly developed phage cocktail targeting *Enterobacter cloacae*, which provides insight into the critical role of administration doses and timing in optimizing phage therapy for CRECC infections and offers a promising strategy for combating AMR.

## MATERIALS AND METHODS

### Bacterial strains and culture conditions

In this study, bacterial strains were obtained from the clinical sample repository of the Second Hospital of Nanjing, as listed in Table S1. The strains included *Acinetobacter baumannii*, *E. cloacae* complex species, *Escherichia coli*, *Klebsiella pneumoniae*, *Pseudomonas aeruginosa*, and *Staphylococcus aureus*. All strains were cultured on Luria-Bertani (LB) agar or in LB broth at 37°C. When necessary, apramycin was added at a concentration of 100 µg/mL to ensure plasmid stability.

To confirm the identity of the clinical isolates, bacteria were further characterized both by 16S rDNA sequencing and matrix-assisted laser desorption ionization-time of flight (MALDI-TOF) mass spectrometry using a Clin-TOF-II instrument (BioYong, China), following the manufacturer’s recommended procedures.

### Isolation, purification, and host range of phages

Phages were isolated and purified from sewage using a double-layered plating method as previously described (15). Briefly, untreated sewage samples were collected from the Public Hygiene and Medical Center of Nanjing. The samples were pretreated by filtration through a 0.22 µm membrane to remove environmental bacteria. Thirty-seven *Enterobacter* species strains were used as bacterial hosts. To isolate phages, 100 µL of the filtered sewage was mixed with 500 µL of bacterial culture, combined with 0.8% warm agar supplemented with 2 mM CaCl₂, and poured onto LB plates containing 2.5% agar. The plates were incubated at 37°C for 12 h, and plaque formation was monitored. A single plaque was selected for enrichment and further purified through six rounds of the double-layer plate method. The purified phage was stored at 4°C for routine use and at −80°C for long-term preservation.

Phage sensitivity of the bacterial strains was assessed using a spot assay (16). In brief, 500 µL of log-phase bacterial culture was mixed with soft agar and poured onto LB agar plates. After cooling and solidification, 5 µL of each phage solution was spotted on the surface of the plates. The plates were incubated at 37°C for 12 h, and phage spots were examined. A total of 37 *Enterobacter* species strains were tested, along with a few randomly selected bacterial strains, including *A. baumannii*, *E. coli*, *K. pneumoniae*, *P. aeruginosa*, and *S. aureus*.

### CsCl gradient ultracentrifugation and transmission electron microscopy

To observe phage morphology, isolated phages were further purified using density gradient centrifugation with cesium chloride (CsCl), as previously described (17). Briefly, the phage solution was gradually added to the CsCl gradient and centrifuged in a Beckman X-100 ultracentrifuge equipped with a SW 41 Ti rotor at 120,000 × *g* for 2 h at 4°C. The supernatant was then collected from the appropriate density layer and transferred to a dialysis membrane (Rui Da Heng Hui, MWCO 5 kDa) for overnight dialysis in 1× SM buffer (Sango Biotech). Finally, the purified phages were negatively stained with 2% uranyl acetate and observed using a Hitachi HT 7700 112 transmission electron microscope (TEM) at Nanjing Agricultural University.

### Bioinformatics analysis

Genomic DNA from the phages was isolated using the Rapid Nucleotide Acid Isolation Kit (BioPerfectus, SDKF60101) on BioPerfectus nucleic acid extraction instruments. The quality of the extracted DNA was assessed by agarose gel electrophoresis and subsequently sent for sequencing on the Illumina NovaSeq 6000 platform at Beijing Novogene Co., Ltd. *De novo* assembly of the clean data was performed using SPAdes v4.0.0 (18). Host genome sequences were eliminated through BLAST analysis utilizing Bowtie2 v2.5.4 (19). The circular or linear nature of the phage genomes was confirmed by PCR with primers specific to the genomic termini, followed by Sanger sequencing (Table S1).

Phage genomes were annotated using Prokka 1.14.6 (20) and analyzed for GC content using GC View (21). Genomic organization and comparison were analyzed with Easyfig (22). Virulence factor analysis was conducted using the Virulence Factor Database (VFDB) (23), whereas antibiotic resistance was assessed using the Comprehensive Antibiotic Resistance Database (CARD) (24). A phylogenetic tree was constructed based on the capsid protein or the large subunit terminase protein using MEGA 7 (25). The complete genomic sequences of ФEBU8, ФECL22, and ФECL30 have been deposited in the GenBank database under accession numbers PQ227828, PQ227830, and PQ227829, respectively.

SNP/Indel variations between the wild-type strain *E. cloacae* 22 and its spontaneous phage-resistant mutants were analyzed. Bacteria were cultured to the log phase, and genomic DNA was extracted using a DNA extraction kit. A genomic library was then constructed, followed by PE150 sequencing on the Illumina platform. Sequence alignment analysis was performed by comparing the sequencing data against the reference genome using BWA (v0.7.17) (26) and SAMtools (v1.7) (27). The genomic sequences of *E. cloacae* 22, R3, R4, and R6 have been deposited in the NCBI database with accession number PRJNA1175430.

### Determination of the biological features of phage

To evaluate the pH stability of phage ФECL22, citrate-phosphate buffer was prepared for pH values ranging from 3.0 to 8.0, and Tris-HCl buffer was prepared for pH values from 9.0 to 12.0. A total of 100 µL of phage was mixed with 900 µL of the corresponding buffer at different pH levels and incubated for 1 h. After incubation, the phage titers were determined using the double-layer agar plate method (28, 29). The titer in the pH 7.0 buffer served as a control, and the remaining phage counts in the different pH buffers were normalized to this control group. The experiments were repeated three times.

To assess the stability of phage ФECL22 at different temperatures, 500 µL of phage in 1× SM buffer was incubated at various temperatures for 1 h, after which the phage titer was determined using the double-layer agar plate method. Phage incubated at 25°C was set as the control, and the remaining phage counts at different temperatures were normalized to the control group. These experiments were also performed in triplicate.

Phage sensitivity to UV light was determined as described previously (30). Phages in 1× SM buffer were exposed to UV light, and the samples were taken at different time points to assess phage viability using the double-layer agar plate method. These experiments were performed in triplicate.

To evaluate the adsorption capacity of ФECL22 on *E. cloacae* 22, the log-phase culture of *E. cloacae* 22 was prepared, washed, and suspended in fresh LB broth. A mixture of 500 µL of 1 × 10^7^ PFU/mL ФECL22 was combined with 5 mL of *E. cloacae* culture (1 × 10⁸ CFU/mL) and incubated at 37°C with shaking. The samples (100 µL) were taken at various time intervals, and the remaining phage titers in the supernatant were measured and normalized to the initially added phage count. The experiments were performed in triplicate.

To determine the optimal multiplicity of infection (MOI) of phage ФECL22 against *E. cloacae* 22, the log-phase culture of *E. cloacae* 22 was prepared, washed, and suspended in fresh LB broth. Five hundred microliters of ФECL22 solution with different doses was mixed with 5 mL of *E. cloacae* culture (1 × 10⁸ CFU/mL) and incubated at 37°C with shaking overnight. The phage titers in the supernatant were measured. The MOI producing the highest phage titer was considered the optimal MOI for the phage (31). The experiments were performed in triplicate.

The one-step growth curve of phage ФECL22 was measured according to a previously established protocol with minor modifications (32). To measure this growth curve, the log-phase culture of *E. cloacae* 22 was prepared, washed, and suspended in fresh LB broth. Five hundred microliters of 1 × 10⁵ PFU/mL ФECL22 was combined with 500 µL of 1 × 10⁷ CFU/mL *E. cloacae* culture at an MOI of 0.01. After incubation for 10 min at 37℃, the mixture was centrifuged at 12,000 rpm for 1 min, washed once, suspended in 1 mL of pre-warmed LB, and then transferred to 100 mL of pre-warmed LB. This time point was designated as 0. Samples (1 mL) were taken at different time points, and phage titers in the supernatant were measured. The burst size was calculated using the formula: total number of phages liberated at the end of one growth cycle divided by the number of infected bacteria. These experiments were performed in triplicate.

### Plasmid construction and transformation

To construct the OmpA expression plasmid, pBECAb-Apr (33) was linearized by reverse PCR using primers BECAb-qR and BECAb-bF. This linearized plasmid was then fused with an araC-pBAD promoter fragment, amplified from pHERD20T using primers araC-F and araC-R, through the In-Fusion cloning method, generating pWCab24T. The *ompA* gene was amplified from *E. cloacae* 22 and *E. coli* Stellar (TaKaRa Bio) by PCR with primers ompA-F and ompA-Ecl-R2 or ompA-Eco-R2, respectively. These fragments were inserted into pWCab24T, digested with EcoRI and HindIII, via In-Fusion, yielding pWCab24T-OmpA_ECL_ and pWCab24T-OmpA_ECO_. Sequencing confirmed the correctness of these plasmids.

Electroporation-competent cells of *E. cloacae* and *A. baumannii* were prepared as described previously (33). Briefly, their log-phase cultures were first prepared in LB broth. For *E. cloacae*, 5 µg/mL polymyxin B nonapeptide (PMBN) was added to enhance transformation efficiency (34). Cultures were washed twice with cold 10% glycerol and resuspended in 10% glycerol. Competent cells of *K. pneumoniae* 84 were prepared following the established protocols (35), with cultures harvested directly from agar plates to reduce capsular polysaccharides and then washed twice with cold water. For transformation, 500 ng of plasmid DNA was used, and electroporation was conducted using a Gene Pulser Xcell Electroporation System under conditions of 2.5 kV, 25 µF, and 200 Ω. To induce OmpA expression, the cultures were supplemented with 0.2% arabinose for each transformant.

### *In vitro* bactericidal activity

To investigate the *in vitro* bactericidal activity of phage ФECL22, a log-phase culture of *E. cloacae* 22 was washed and suspended in fresh LB broth and dispensed into a 96-well plate, with each well containing 100 µL of suspension at 1 × 10^6^ CFU. Various concentrations of purified ФECL22 phage solution, obtained through ultracentrifugation, were added to the wells at different MOI. Bacteria without phage served as a growth control, and LB broth alone was used to check for contamination. Each MOI was tested in eight replicate wells. Bacterial growth was monitored over a 24 h period by measuring the optical density at 600 nm (OD₆₀₀) every hour. The experiments were performed in triplicate.

To evaluate the bactericidal efficiency of the phage cocktail, ФEBU8, ФECL22, and ФECL30 were mixed at a 1:1:1 ratio. Equal volume of the bacterial suspension (1 × 10⁶ CFU) and the phage cocktail (1 × 10⁶ PFU) were added to the 96-well plate. Eight replicate wells were set up. Each single phage solution was used as a control. Bacterial growth was monitored as above, and the experiments were repeated three times.

### CCK8 assay

Cytotoxicity of phage suspensions against LO2 in normal hepatocytes was assayed using the Cell Counting Kit 8 (CCK8, Solarbio, CA1210) according to the manufacturer’s manual. Briefly, LO2 cells were inoculated in DMEM medium containing 10% FBS in 96-well plates with 5 × 10^3^ cells per well and incubated for 24 h at 37°C in a 5% CO2 environment. Then, the cocktail suspension was diluted with a gradient of DMEM medium and added to 96-well plates for 24 h of incubation. CCK8 solution was added to each well and incubated at 37°C for 45 min, and OD_450_ was measured using an enzyme marker. The experiment was repeated three times.

### Safety evaluation of phage cocktail *in vivo*

In this study, specific pathogen-free (SPF) Kunming (KM) female mice, aged 7–8 weeks and weighing 30–34 g, were utilized for all experimental procedures. The mice were obtained from Spearfish (Suzhou) Biotechnology Co. and allowed a 1-week acclimatization period, during which they were provided free access to food and water and maintained under a standard light/dark cycle at room temperature.

To assess the *in vivo* safety of the phage cocktail, the mice were randomly assigned to five groups, with five mice in each group. The phage cocktail was formulated by combining purified phages ФEBU8, ФECL22, and ФECL30 in a 1:1:1 ratio, subsequently diluted to the desired concentrations in 1× PBS. The phage suspensions were administered via two routes: intraperitoneally (IP) and intravenously (IV). For the IP group, three dosage levels were evaluated: 1 × 10⁸ PFU/mouse, 1 × 10⁶ PFU/mouse, and 1 × 10⁴ PFU/mouse. The IV group received a single dosage of 1 × 10⁸ PFU/mouse. Each mouse received 200 µL of phage suspension daily for a duration of 20 days.

Clinical observations and body weight measurements were recorded daily by two trained observers, commencing at the time of injection. A scoring system adapted from the Mouse Sepsis Scoring Scale (MSS) was employed (36), with a modified scoring framework where a score of 5 was assigned for dead mice and a score of 0 for healthy individuals. At the end of the study, orbital blood samples were collected for the evaluation of endotoxin levels and cytokine profiles, and mice were humanely euthanized via cervical dislocation.

### Endotoxin measurement

Endotoxin levels in mouse blood were measured using the Toxinsensor™ Chromogenic LAL Endotoxin Assay Kit (L00350C) from GenScript (China). Standards were prepared according to the manufacturer’s instructions, and three groups were established: a standard group, a sample group, and a negative control group using pyrogen-free water. In each tube, 100 µL of standard, 100 µL of blood sample, and 100 µL of pyrogen-free water were added, followed by the corresponding reagents from the kit. The mixture was then transferred to a 96-well plate, and the absorbance was measured at 545 nm using a microplate reader. A standard curve was constructed based on the OD_545_ values from the standards, allowing for the calculation of endotoxin content in the samples.

### Inflammatory factor measurement

Inflammatory factors TNF-α, IL-1β, and IL-6 in mouse serum were quantified using specific ELISA kits: the Mouse IL-1β ELISA Kit (EMC001b), Mouse TNF-α ELISA Kit (EMC102a), and Mouse IL-6 ELISA Kit (EMC004), all obtained from Quanticyto (China). Standards were prepared according to the manufacturer’s instructions. Each assay included a standard group, a sample group, and a blank dilution serving as a negative control. In each test tube, 100 µL of standard, 100 µL of plasma sample, and 100 µL of blank dilution were added, followed by the corresponding reagents from the kits for incubation. The mixtures were then transferred to a 96-well plate, and absorbance was measured at 450 nm using a microplate reader. Standard curves were established based on the OD_450_ values from the standards, allowing for the calculation of TNF-α, IL-1β, and IL-6 levels in the samples.

### Establishment of bacteremia mouse model infected by *E. cloacae*

To explore the conditions for establishing a successful bacteremia model caused by *E. cloacae* infection, 8-week-old female KM mice were divided into four groups, with five mice in each group. The log-phase culture of *E. cloacae* 22 was collected, washed, and suspended in 1× PBS. A total of 200 µL of the bacterial culture was injected intraperitoneally into each mouse at concentrations of 1 × 10⁸ CFU/mL, 1 × 10⁹ CFU/mL, and 1 × 10^10^ CFU/mL, respectively. An equal volume of PBS served as a negative control. Two researchers monitored the clinical conditions of the mice every 2 h for the first 12 h and then once daily. The scoring system used was adapted from murine sepsis score criteria (36), with modifications: a dead mouse was assigned a score of 5, whereas a healthy mouse received a score of 0. Kaplan-Meier survival curves were analyzed using GraphPad Prism 9.3.0.

### Evaluation of treatment efficacy of phage cocktails with various doses

To investigate the therapeutic effects of varying doses of phage cocktail on bacteremia infection, mice were randomly assigned to five groups, each consisting of eight mice. A bacteremia model was first established by intraperitoneally injecting 200 µL of 1 × 10^10^ CFU/mL *E. cloacae* 22. Mice receiving an equal volume of 1 × PBS served as an uninfected control. At 1 h POI, the phage cocktail was administered intraperitoneally at different doses: 1 × 10⁸ PFU/mouse (high dose), 1 × 10⁶ PFU/mouse (middle dose), and 1 × 10⁴ PFU/mouse (low dose). An equal volume of 1 × PBS served as a negative control. Clinical conditions of the mice were monitored by two researchers every 2 h for the first 12 h and then once daily for the entire 168-h duration. Kaplan-Meier survival curves were analyzed using GraphPad Prism 9.3.0. Body weight was also recorded daily.

To assess treatment efficacy, a checkpoint was established at 24 h POI. Three mice were randomly selected from each group, euthanized, and dissected. Blood, liver, and spleen samples were collected for analysis of phage titer and bacterial load. Six colonies were randomly selected from cultured plates for blood, liver, and spleen samples across the three different doses, and their susceptibility to the phage cocktail was evaluated using the spot test.

### Evaluation of treatment efficacy of phage cocktails with various timings

To investigate the effects of phage cocktail treatment at various time points, mice were randomly assigned to eight groups, each consisting of 13 mice. A bacteremia mouse model was established as described previously, with mice receiving an equal volume of 1× PBS serving as an uninfected control. Infected mice were treated with the phage cocktail at a dose of 1 × 10⁶ PFU/mouse at different time points: 24 h prior to infection (“−24”), 6 h prior to infection (“−6”), immediately upon infection (“0”), 1 h POI (“1”), 6 h POI (“6”), and a combination of treatment at 24 h prior and 6 h post-infection (“−24 + 6”). An equal volume of 1× PBS was used as a negative control. Clinical conditions of the mice were monitored by two researchers every 2 h for the first 12 h and then once daily for the entire 168-h duration. Kaplan-Meier survival curves were analyzed using GraphPad Prism 9.3.0. Body weight was also recorded daily.

To assess treatment efficacy, a checkpoint was established at 24 h POI. Three mice were randomly selected from each group, euthanized, and dissected. Blood, liver, and spleen samples were collected for analysis of phage titers, bacterial loads, and histopathological examination. Blood samples were also used to evaluate cytokine profiles and perform routine hematological examinations, which were conducted using the Sysmex XN-2800 hematology analyzer.

### Histopathological examination

Tissue sections from the livers, lungs, kidneys, and spleens were fixed in 4% paraformaldehyde, embedded in paraffin, and stained with hematoxylin and eosin (H&E). Microscopic examination was conducted to evaluate the histological features of the tissues.

### Statistical analysis

Statistical analysis was conducted using GraphPad Prism 9.3.0 software. A one-way ANOVA with multiple comparisons was used to assess differences among groups. Student’s *t*-test was applied for comparisons between the two groups, and the log-rank test was used to analyze differences in survival curves. Statistical significance was defined as *P* < 0.05.

## RESULTS

### Most isolated *Enterobacter* phages exhibit broad host spectra

**Fig 1:**
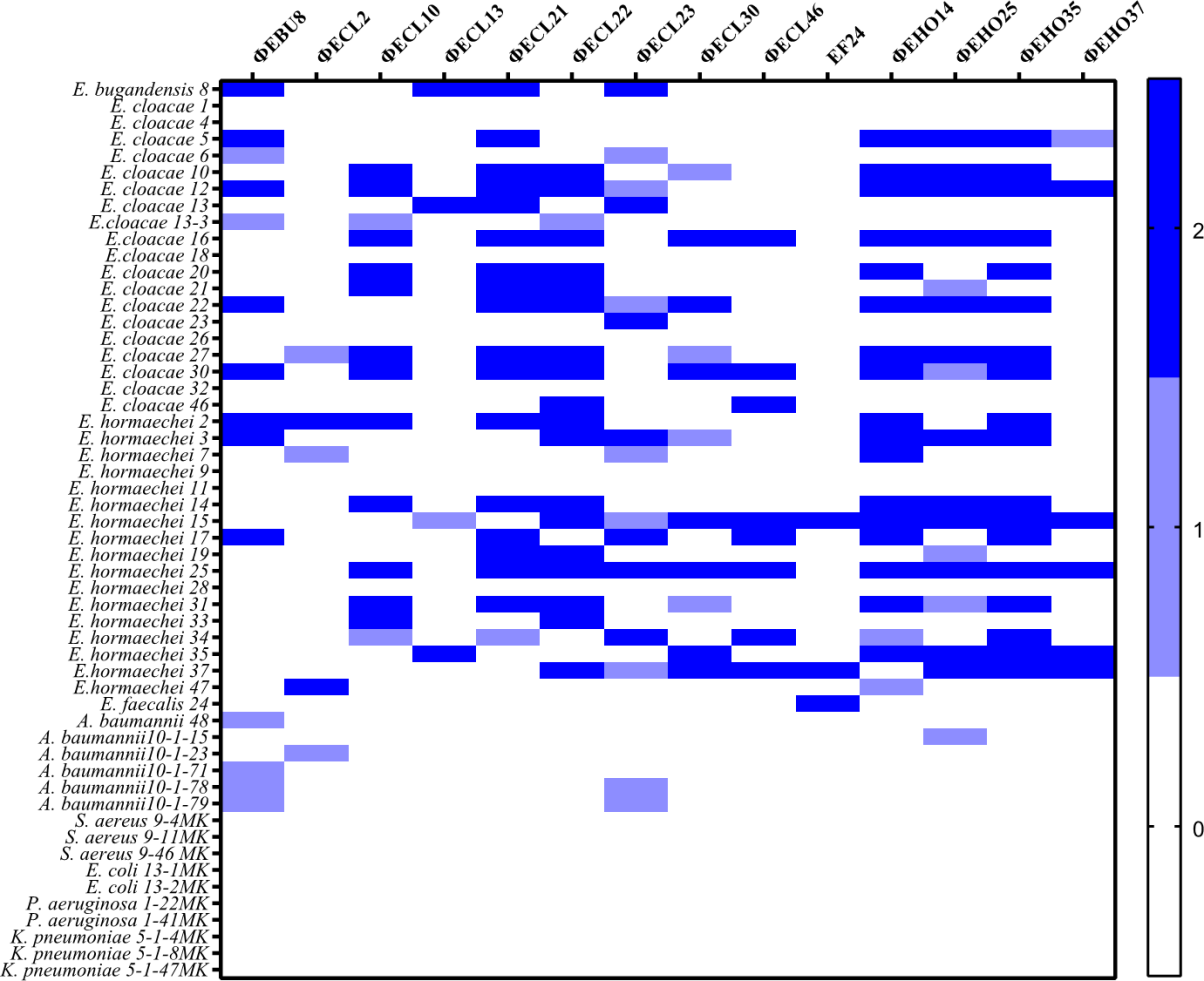
Heatmap of host spectra of isolated *Enterobacter* phages. Host ranges of phages were evaluated using a spot test against clinical bacterial strains. Dark blue indicates large and clear plaques, light blue represents vague plaques, and white signifies no plaque formation.

To isolate lytic phages, 38 clinical isolates from the *Enterobacter cloacae* complex species were screened using untreated hospital sewage. A total of 14 lytic phages were successfully isolated (Fig. 1). Molecular identification, based on 16S rDNA sequencing and mass spectrometry, revealed that the *E. cloacae* isolates consisted of 1 *Enterobacter bugandensis* (EBU), 19 *Enterobacter cloacae* (ECL), and 17 *Enterobacter hormaechei* (EHO) strains, as well as 1 *Enterococcus faecalis* (EF) strain.

Spot assays revealed varied host ranges among the isolated *Enterobacter* phages (Fig. 1). Notably, the majority of the isolated phages were capable of lysing both *E. cloacae* and *E. hormaechei*. In detail, phages ФECL2, ФECL13, and ФEHO37 exhibited relatively narrow host spectra, lysing 10.8%, 10.8%, and 16.2% of the 37 tested ECC strains, respectively. In contrast, phages ФECL21, ФECL22, ФEHO14, and ФEHO35 demonstrated broader host ranges, lysing 48.6%, 51.3%, 51.3%, and 48.6% of the ECC strains, respectively (Fig. S1). Interestingly, *Enterobacter* phages ФEBU8, ФECL23, and ФEHO25 showed lytic activity against certain *A. baumannii* strains. However, the efficiency of plating (EOP) of ФEBU8 and ФECL23 against *A. baumannii* 10-1-78 was significantly lower compared with their original host strains. Growth of this *A. baumannii* strain was inhibited in the presence of these phages (Fig. S2). Additionally, *Enterococcus* phage ФEF24 demonstrated the ability to lyse *E. hormaechei*. None of the isolated phages exhibited lytic activity against other tested pathogens, including *E. coli*, *K. pneumoniae*, *P. aeruginosa*, or *S. aureus*.

**Fig 2:**
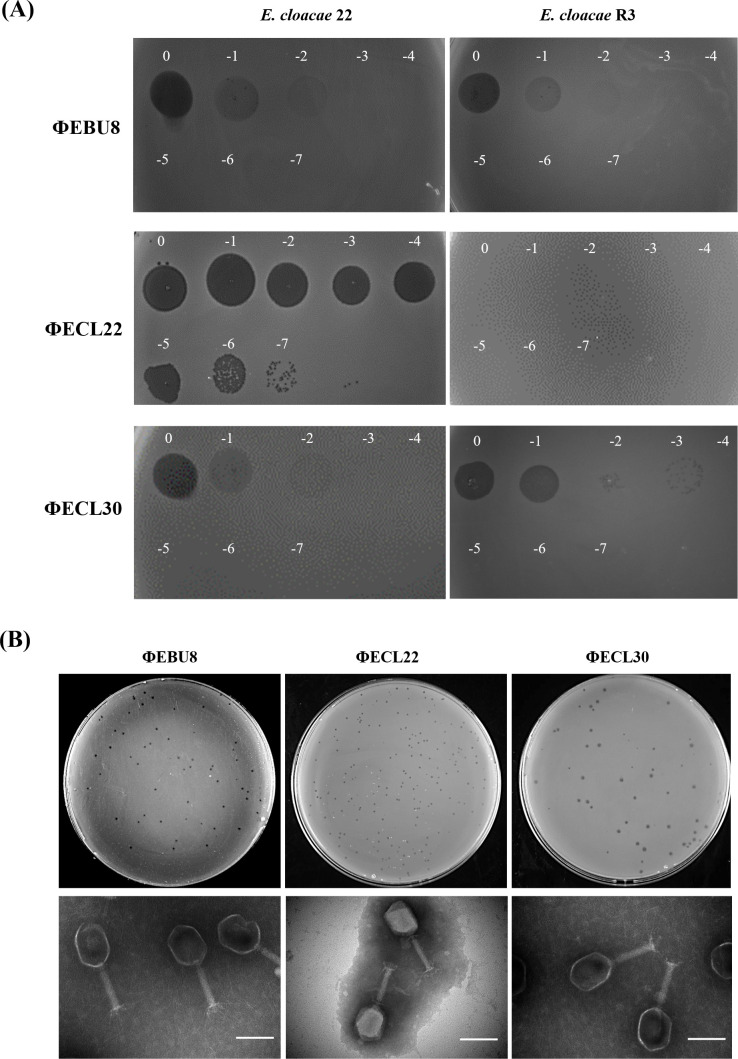
Morphologies of ФEBU8, ФECL22, and ФECL30. (**A**) Lysis test of ФEBU8, ФECL22, and ФECL30 against *E. cloacae* 22 and its derivative ФECL22-resistant mutant R3. A concentration of 1 × 10⁹ PFU/mL of each phage was used for the spot assay. (**B**) Phage plaques of three phages on LB agar with their respective bacterial hosts (top), and their transmission electron microscopy (TEM) images (bottom). Magnification: ×60,000; scale bar: 100 nm.

Among the isolated phages, ФEBU8, ФECL22, and ФECL30 were selected for further characterization for three reasons. First, they exhibited relatively wide but distinct host ranges. Second, all three were able to lyse *E. cloacae* 22, and both ФEBU8 and ФECL30 could lyse the ФECL22-resistant mutant R3 derived from *E. cloacae* 22 (Fig. 2A). *E. cloacae* 22 is a carbapenem-resistant clinical isolate from cerebrospinal fluid and was chosen for establishing a bacteremia mouse model in this study. Third, their combination effectively lysed 86.5% (32/37) of *E. cloacae* complex strains and 66.7% (4/6) of *A. baumannii* strains (Fig. 1). These three phages formed very small, transparent pinhole plaques without halos (Fig. 2B, Top), indicating that these phages might not have depolymerase activity. Transmission electron microscopy (TEM) revealed that all three phages exhibited the typical structure of *E. coli* T4 phages, characterized by an elongated icosahedral head, a tail, and long tail fibers (37) (Fig. 2B, Bottom). The contractile tail structure is functionally significant, as it facilitates efficient DNA injection into bacterial cells, which may contribute to the phage’s high lytic efficiency observed in this study. Consequently, they were classified within the Myoviridae family and designated as vB_EbuM-8, vB_EclM-22, and vB_EclM-30, respectively. For simplicity, they are referred to as ФEBU8, ФECL22, and ФECL30 throughout.

### ФEBU8, ФECL22, and ФECL30 are novel *Enterobacter* phages with different genomic organizations and evolution histories

**Fig 3:**
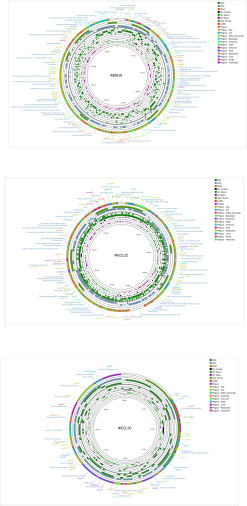
Genomic characteristics of three *Enterobacter* phages. Circular representations of the phage genomes. The icon in the upper right corner indicates that different color blocks represent various annotation contents. The outer circle displays seven tracks: (i) Phigaro: predicted prophage regions; (ii) CARD: prediction of resistance genes; (iii) Alien Hunter: events of putative horizontal gene transfer (HGT); (iv) CDS: functional genes annotated using the Bakta method; (v) ORFs: orientation of transcribed genes, indicated as clockwise or anticlockwise; (vi) GC content: percentage of guanine and cytosine (G + C) content; and (vii) GC skew: values greater than zero are shown in green, whereas values less than zero are depicted in magenta.

The complete genomes of phages ФEBU8, ФECL22, and ФECL30 were found to be 172,768 bp, 177,652 bp, and 40,126 bp in length, respectively, with GC contents of 39.69%, 44.71%, and 52.09%, respectively (Fig. 3). PCR using terminal primers (Table S1) confirmed that ФEBU8 and ФECL30 are linear, whereas ФECL22 is circular. ФEBU8 was annotated to contain 298 coding sequences (CDS) and 18 tRNA genes, ФECL22 had 271 CDS, and 2 tRNA genes, whereas ФECL30 had 48 CDS and 1 tRNA gene (Table S2).

The complete genome of ФEBU8 shared 94% coverage and 96.83% identity with *Enterobacter* phage vB_Ent31, and 93% coverage with 88.81% identity with *Enterobacter* phage vB_EluP_RZH. ФECL22 exhibited 97% coverage and 99.72% identity with *Enterobacter* phage phi5, and 93% coverage with 91.58% identity with *Enterobacter* phage ENC20. Additionally, ФECL30 showed 97% coverage and 98.92% identity with *Enterobacter* phage IME278, and 85% coverage with 88.82% identity with *Enterobacter* phage EP1. A linear comparison of these three phage genomes with their respective homologous phages revealed similar sequences but distinct genomic organizations, suggesting that recombination events may have occurred (Fig. S3). Phylogenetic analysis based on the major capsid protein and the large subunit of the terminase indicated that ФEBU8, ФECL22, and ФECL30 belonged to distinct clades, although each shared a close relationship with different *Enterobacter* phages (Fig. S4).

Furthermore, analysis using VFanalyzer revealed no virulence genes present in the genomes of these three phages, and Comprehensive Antibiotic Resistance Database-RGI analysis did not detect any antibiotic resistance genes. Therefore, these three phages represent novel *Enterobacter* lytic phages with potential suitability for therapeutic applications.

### Characterization of the biological features of phage ФECL22

**Fig 4:**
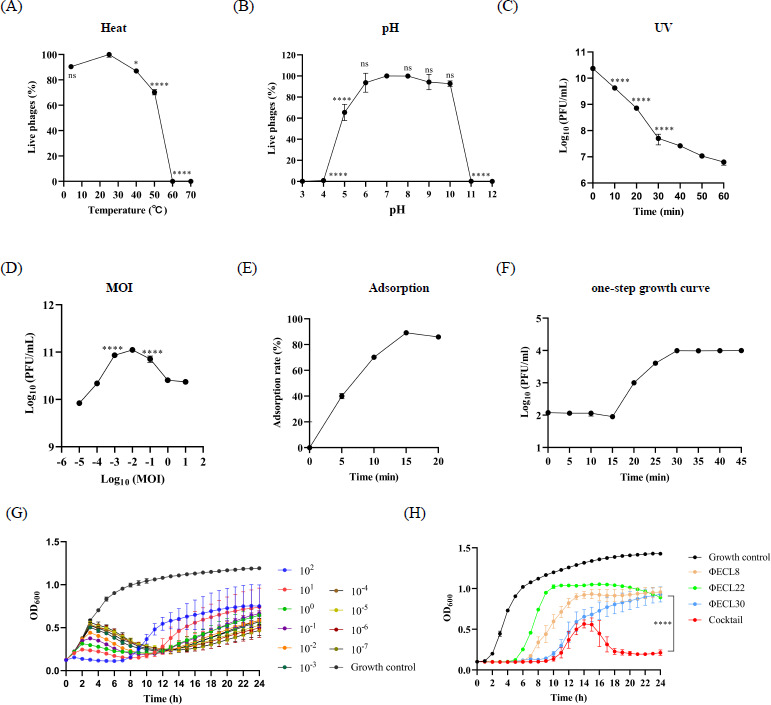
Biological characteristics of ФECL22. (**A**) Heat tolerance: the phage viability at 25°C was set to 100%. Statistical differences between other temperature groups and the 25°C group were analyzed using one-way ANOVA with multiple comparisons. (**B**) pH stability: the phage viability at pH 7.0 was set to 100%. Statistical differences between other pH groups and the pH 7.0 group were analyzed using one-way ANOVA with multiple comparisons. (**C**) UV sensitivity: the phage viability at time 0 was set to 100%. Statistical differences between other time points and time 0 were analyzed using one-way ANOVA with multiple comparisons. (**D**) Optimal MOI: the phage titer at MOI 0.01 was set as the control. Statistical differences between other MOI groups and the control were analyzed using one-way ANOVA with multiple comparisons. (**E**) Adsorption rate: phage adsorption efficiency in LB broth was measured over time. (**F**) One-step growth curve: latent and burst phases of ФECL22 were analyzed. (**G**) *In vitro* growth inhibition with different MOI: bacterial growth curves were measured at varying MOIs. (**H**) *In vitro* growth inhibition with a phage cocktail: phage cocktail composed of ФEBU8, ФECL22, and ФECL30 was compared with individual phages. Statistical differences in OD_600_ at 24 h between the cocktail and phage-alone groups were analyzed using student’s *t*-test. Statistical significance: *P < 0.05; ***P < 0.001; ****P < 0.0001; ns, not significant.

Given that ФECL22 exhibited a broad host range, capable of lysing 51.4% (19/37) of *E. cloacae* complex species (Fig. 1), we decided to investigate its biological features as a representative of *E. cloacae* phages. Thermal stability tests indicated that ФECL22 could withstand relatively high temperatures, with over 70% of live phage remaining even at 50°C (Fig. 4A). The lytic activity decreased rapidly beyond this temperature. pH stability tests demonstrated that ФECL22 remained stable within the pH range of 6.0 to 10.0, maintaining over 90% lytic activity in this range (Fig. 4B). Similar to other phages, ФECL22 was sensitive to UV irradiation, with a reduction of 2 log units in phage titer after 20 min of exposure (Fig. 4C).

The adsorption rate measurement revealed that approximately 90% of ФECL22 particles were adsorbed onto *E. cloacae* 22 in LB broth within 15 min (Fig. 4D). ФECL22 had an optimal multiplicity of infection (MOI) of 0.01, producing approximately 1 × 10¹¹ PFU/mL of progeny phage at this MOI (Fig. 4E). The phage exhibited a latent period of 15 min and a prolonged lysis period of 15 min, reaching a plateau after 30 min (Fig. 2F). The average burst size was calculated to be approximately 19 phage particles per infected bacterial cell.

The growth of *E. cloacae* 22 was inhibited by ФECL22 alone within the first 6 h, but bacterial growth rebounded afterward (Fig. 4G). Increasing the number of phages improved the inhibitory effect. Interestingly, although a low MOI did not prevent bacterial growth in the initial phase, the final inhibitory effect after 24 h was comparable with that of a high MOI, likely due to phage proliferation (Fig. 4G). When combined with the other two phages, ФECL22 was able to completely inhibit bacterial growth within 24 h. ФEBU8 and ФECL30 exhibited stronger inhibitory effects than ФECL22, possibly because they could inhibit some ФECL22-resistant mutants (Fig. 4H). Remarkably, there was a brief rebound in bacterial growth in the cocktail-treated group, the mechanism of which warrants further investigation.

### OmpA is the receptor of phage ФECL22

**Fig 5:**
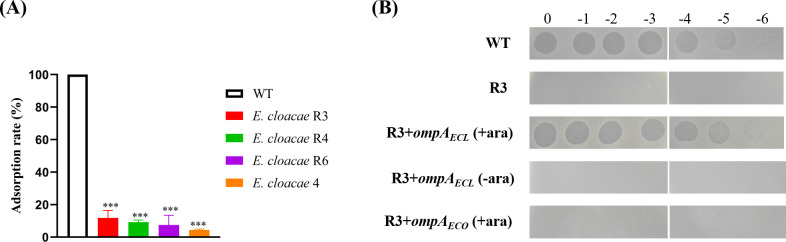
Identification of the phage receptor for ФECL22. (**A**) Adsorption assays demonstrate impaired binding of ФECL22 to the three spontaneous phage-resistant mutants, R3, R4, and R6, as well as the natural ФECL22-resistant *E. cloacae* 4, compared with the wild-type strain, *E. cloacae* 22. Statistical differences among groups were analyzed using one-way ANOVA with multiple comparisons; ***P < 0.001. (**B**) Introduction of OmpA from *E. cloacae* into the R3 mutant restores phage sensitivity to ФECL22.

To identify the receptor of phage ФECL22 in *E. cloacae*, we isolated three spontaneous phage-resistant mutants from *E. cloacae* 22, designated R3, R4, and R6. Adsorption assays indicated that ФECL22 did not attach efficiently to these mutants compared with *E. cloacae* 22 (Fig. 5A), suggesting that their phage resistance was due to impaired adsorption. To compare the genomic differences between the wild-type strain and these mutants, we sequenced their genomes and analyzed their SNP/Indel variations. The mutants R3, R4, and R6 had 125, 127, and 130 SNP sites, respectively, compared with the wild-type strain (Table S3). Notably, both R3 and R4 harbored two termination mutations in the *ompA* gene, resulting in truncated OmpA proteins. Although SNP and indel analysis did not reveal mutations in the *ompA* gene of R6, PCR and Sanger sequencing identified a termination mutation in R6, where the codon for the 82nd tryptophan (TGG) was replaced by a termination codon (TAG). Therefore, given that several T-even-like bacteriophages use OmpA as a receptor in *E. coli* (38), we hypothesized that ФECL22 might also utilize OmpA as its receptor. To test this, we expressed OmpA from *E. cloacae* in the R3 mutant, which restored the bacterium’s sensitivity to ФECL22, making it comparable with the wild-type strain (Fig. 5B). However, when OmpA from *E. coli* was introduced into the R3 mutant, the bacterium remained phage-resistant. These findings suggest that OmpA in *E. cloacae* is the specific receptor for ФECL22, and the *E. coli* version of OmpA could not substitute for its homolog, despite an 86% sequence identity.

To determine if variations in the OmpA protein sequence influence sensitivity to ФECL22 among *E. cloacae* complex species, we randomly selected six ФECL22-resistant and seven ФECL22-sensitive strains and sequenced their *ompA* genes. Sequence analysis revealed almost identical sequences among these strains (Fig. S5). We further examined one ФECL22-resistant strain, *E. cloacae* 4, which had an identical OmpA protein as *E. cloacae* 22. However, an adsorption assay showed that ФECL22 even failed to adsorb on this strain (Fig. 5A). Additionally, introducing *E. cloacae* 22’s OmpA into *E. coli*, *K. pneumoniae*, and *A. baumannii* did not confer sensitivity to ФECL22 (data not shown). These findings suggest that the presence of an intact OmpA alone does not guarantee successful ФECL22 infection, indicating the likely involvement of an auxiliary receptor in *E. cloacae*.

### Phage cocktail is safe both *in vitro* and *in vivo*

**Fig 6:**
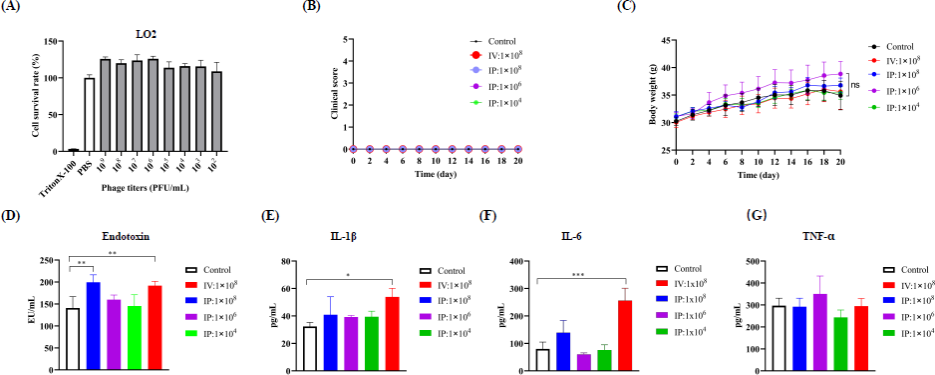
Safety evaluation of the phage cocktail. (**A**) CCK-8 assay demonstrated no cytotoxicity of the phage solution on LO2 cells, with 1 × PBS and 0.1% Triton X-100 serving as negative and positive controls, respectively. (**B**) Assessment of acute toxicity of the phage cocktail in a mouse model, with various doses administered via IP or IV routes over 20 days. Clinical scores were monitored, where “0” indicates healthy status. (**C**) Changes in body weight during the study. (**D**) Measurement of endotoxin levels at the 24-h post-injection checkpoint. (**E-G**) Cytokine levels were measured at the same checkpoint. 1× PBS served as the negative control. Statistical differences among groups were analyzed using one-way ANOVA with multiple comparisons; *P < 0.05, **P < 0.01, ***P < 0.001.

Since *E. cloacae* rapidly developed resistance against a single phage, we investigated the treatment efficacy of a 3-phage cocktail composed of purified ФEBU8, ФECL22, and ФECL30 in a 1:1:1 combination. Before initiating phage treatment, we assessed the cytotoxicity of this phage cocktail. The CCK8 assay demonstrated that the phage cocktail did not exhibit any adverse effects on the human hepatic cell line LO2, even at a high concentration of 1 × 10⁹ PFU/mL (Fig. 6A). This finding indicates that our phage preparation is safe *in vitro*.

Subsequently, we evaluated the *in vivo* safety of the phage cocktail by administering it to mice once daily for 20 days, either via IP or IV injection. Mice were monitored for clinical signs, and health scores indicated that they remained healthy with no obvious symptoms (Fig. 6B). Additionally, the body weight of mice injected with the phage solution did not show significant changes over the 20-day period (Fig. 6C). On the last day post-injection, all mice were sacrificed, and blood samples were collected to measure endotoxin levels and cytokines. Compared with the control group, both IV and IP injections of 1 × 10⁸ PFU per mouse resulted in significantly higher endotoxin levels (Fig. 6D). Notably, IV injections of the high-dose phage led to increased levels of IL-1β and IL-6, but not TNF-α. However, administration of 1 × 10⁶ PFU per mouse did not cause any adverse effects (Fig. 6F to G). Overall, our phage cocktail demonstrated safety both *in vitro* and *in vivo*.

### Phage cocktail rescues mice from *E. cloacae* bacteriemia in a dose-dependent manner

**Fig 7:**
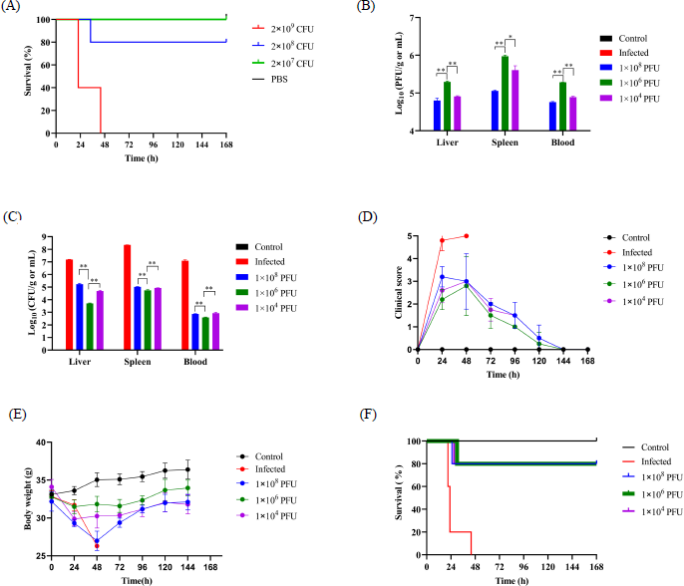
Efficacy of phage cocktail treatment at different doses in a mouse bacteremia model induced by *E. cloacae*. (**A**) Establishment of the mouse bacteremia model through IP infection with varying amounts of *E. cloacae* 22. (**B**) Assessment of bacterial loads in the organs at 24 h POI. (**C**) Measurement of phage titers in the organs at 24 h POI. Statistical differences among groups were analyzed using one-way ANOVA with multiple comparisons; *P < 0.05, **P < 0.01. (**D**) Clinical scoring of treated mice. Student’s *t*-test was applied for comparisons between the high-dose group and the control. ***P < 0.001. (**E**) Changes in body weight over the treatment period. (**F**) Survival curves of infected mice receiving different doses of the phage cocktail.

A bacteremia model was established by intraperitoneally infecting KM mice with varying doses of *E. cloacae* 22. When a dose of 2 × 10⁹ CFU was administered, all five mice died within 44 h post-infection (POI). In contrast, infection with 2 × 10⁸ CFU resulted in only one fatality at 34 h POI, whereas the remaining four mice survived. Mice infected with 2 × 10⁷ CFU displayed no fatalities over a period of 6 days (Fig. 7A). Consequently, a dose of 2 × 10⁹ CFU per mouse was selected to establish the acute infection model.

We subsequently investigated the protective effects of different doses of the phage cocktail administered 1 h POI, including high (1 × 10⁸ PFU/mouse), middle (1 × 10⁶ PFU/mouse), and low (1 × 10⁴ PFU/mouse) doses. After 24 h POI, three mice from each group were sacrificed to measure phage and bacterial loads in the liver, spleen, and blood. Administration of the phage cocktail resulted in abundant phage distribution across these organs. Notably, the middle dose led to the highest phage titers in the liver, spleen, and blood (Fig. 7B); specifically, 9.4 × 10⁵ PFU/g was detected in the spleen of mice treated with the middle dose, compared with 1.1 × 10⁵ PFU/g in the high-dose group and 4.1 × 10⁵ PFU/g in the low-dose group. Consistently, the middle dose also significantly reduced bacterial loads in the liver, spleen, and blood compared with the other two groups (Fig. 7C). In total, 54 bacterial strains were isolated from mice treated with the phage cocktail, all of which were found to be sensitive to it (Fig. S6).

Compared with untreated mice, the treated mice exhibited milder initial symptoms and gradually recovered after 48 h, ultimately returning to an active and healthy state comparable with uninfected controls (Fig. 7D). Body weight data corroborated these observations, indicating that treated mice began to regain weight after 48 h (Fig. 7E). However, it is noteworthy that administration of a high-dose phage cocktail (1 × 10⁸ PFU) resulted in the most severe symptoms and weight loss during the initial 48 h, whereas the middle dose (1 × 10⁶ PFU) led to milder symptoms and a quicker recovery. At the 168-h endpoint POI, 80% (4/5) of the infected mice treated with the phage cocktail survived, regardless of the phage dose (Fig. 7F). Those findings suggest that although the final outcomes were similar across treatment groups, the recovery trajectories were dose-dependent, with the middle dose regimen demonstrating particular efficacy.

### Prophylactic administration offers an opportunity for delayed treatment to rescue bacteremia

**Fig 8:**
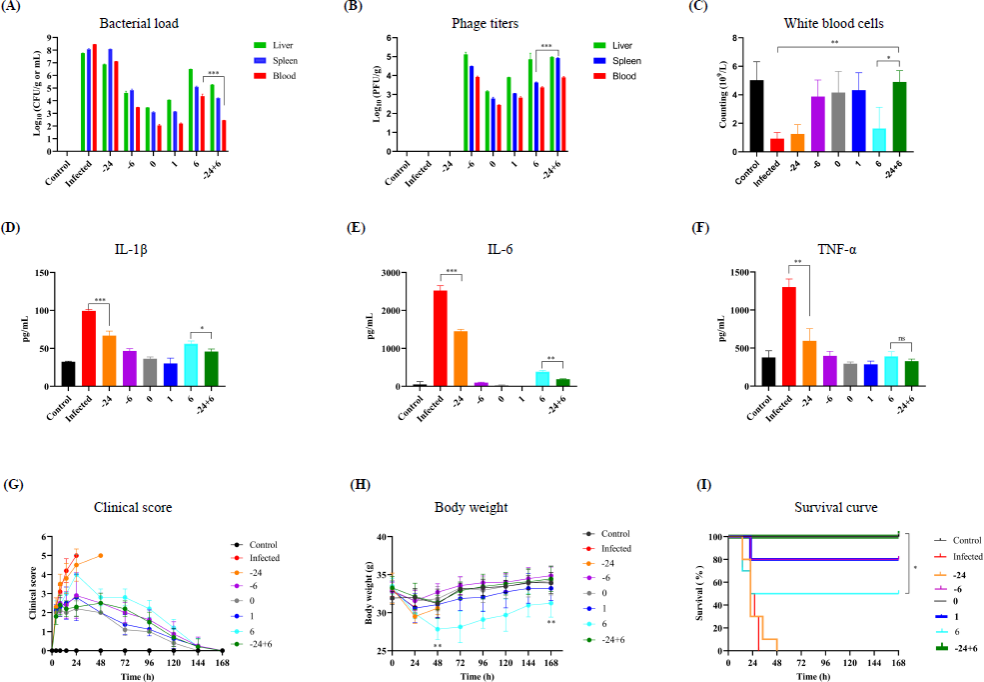
Efficacy of phage cocktail treatment at different time intervals in a mouse bacteremia model induced by *E. cloacae*. (**A**) Assessment of bacterial loads in the organs at 24 h POI. (**B**) Measurement of phage titers in the organs at 24 h POI. (**C**) White blood cell counts determined by routine blood examination at 24 h POI. (**D-F**) Cytokine levels measured at the same checkpoint. Statistical differences among groups were analyzed using one-way ANOVA with multiple comparisons; *P < 0.05, **P < 0.01, ***P < 0.001, ns indicates no significant difference. (**G**) Clinical scores. (**H**) Changes in body weight over the treatment period. Student’s *t*-test was applied for comparisons between the “6” group and the control. **P < 0.01. (**I**) Survival curves of infected mice receiving the phage cocktail at various time intervals, analyzed by the Kaplan-Meier method with statistical significance evaluated using the log-rank test.

To determine the optimal timing for administering the phage cocktail, we evaluated its effects at various intervals: 24 h prior to infection (“−24”), 6 h prior (“−6”), immediately upon infection (“0”), 1 h post-infection (“1”), 6 h post-infection (“6”), and a combination of 24 h prior and 6 h post-infection (“−24 + 6”). At 24 h post-infection, three mice from each group were sacrificed to assess phage and bacterial loads, cytokine levels, and white blood cell counts in the liver, spleen, and blood. Mice treated with the phage cocktail at “−6,” “0,” and “1” displayed significantly lower bacterial loads compared with untreated controls, with earlier treatments yielding better outcomes. Notably, the “−24 + 6” regimen was superior to the “6” regimen, showing markedly reduced bacterial levels in organs (Fig. 8A). Interestingly, no residual phages were detected in the organs of the “−24” group, suggesting complete clearance of phages by 24 h post-administration (Fig. 8B).

Routine blood tests revealed that although *E. cloacae* infection reduced white blood cell counts, phage cocktail treatment restored these levels. The “−24 + 6” regimen, in particular, showed significantly higher white blood cell counts than the “6” regimen (Fig. 8C). Furthermore, the “−24 + 6” regimen led to substantially lower levels of inflammatory cytokines IL-1β, IL-6, and TNF-α compared with the “6” regimen, indicating a stronger anti-inflammatory effect (Fig. 8D through F). Meanwhile, the “6” regimen resulted in the most severe symptoms and significantly lower body weights than the control group, whereas the “−24 + 6” regimen was notably more effective, with improved symptoms and body weights. These findings underscore the enhanced therapeutic potential of the “−24 + 6” regimen over the “6” regimen.

Hematoxylin and eosin (HE) staining of untreated infected mice revealed significant pathological changes, including atrophy of the glomeruli and interstitial congestion. The lungs displayed areas of patchy hemorrhage within the alveolar spaces, whereas the spleen exhibited hemorrhage and congestion in the medullary region, with a clear distinction between the medulla and cortex. Liver cells showed mild swelling and vascular congestion. In comparison to untreated infected mice, the “−24” treatment regimen did not significantly alleviate these symptoms. However, after treatment with the “−24 + 6” regimen, notable improvements were observed: the boundaries between the splenic cortex and medulla became less distinct, medullary congestion was reduced, and cortical hyperplasia was evident, with nearly complete resolution of necrosis within the cortex. Additionally, the areas of alveolar hemorrhage in the lungs showed considerable absorption, glomeruli exhibited slight swelling, and liver cells displayed more pronounced swelling alongside vascular congestion in the interstitium. This level of recovery was superior to that achieved with the “6” regimen (Fig. S7).

At the 168 h endpoint POI, administration at 1 h POI saved 80% (8/10) of the infected mice (Fig. 8G). When treatment was initiated immediately after bacterial challenge, 100% (10/10) survival was observed. However, delaying treatment to 6 h POI significantly reduced efficacy, resulting in only 50% (5/10) survival. Prophylactic administration of the phage cocktail at 24 h prior to infection (“−24”) provided no protective effect, resulting in the death of all infected mice. Notably, the combined administration of “−24 + 6” achieved a 100% survival rate (10/10) (Fig. 8G). Overall, these findings underscore the critical importance of early treatment in managing *E. cloacae* bacteremia with the phage cocktail and prophylactic administration may offer an opportunity for delayed treatment to rescue bacteremia.

## DISCUSSION

The global crisis of antimicrobial resistance has reinvigorated interest in phage therapy (39). However, one of the major challenges for the therapeutic application of phages is their narrow host range. Most previously reported phages are either species- or strain-specific, meaning they can only recognize and lyse a subset of strains within a particular bacterial species (40). Although some researchers have argued that a narrow host range is beneficial, as it reduces the risk of disrupting the human microbiome, there is a concerted effort to identify naturally occurring broad-host range phages or to enhance the host range through phage engineering (41, 42). Phages with broader host ranges are particularly desirable as they can target and eliminate a wider spectrum of pathogenic strains or even related species. *Enterobacter* phages, in particular, are known for their broad host ranges that often cross species boundaries (43). For example, *Enterobacter* phage myPSH1140 was able to lyse 11/15 *E. cloacae*, 2/4 *E. hormaechei*, 2/4 *Enterobacter asburiae*, and 2/3 *Enterobacter aerogenes* isolates (44). Similarly, *E. cloacae* phage ZX14 was able to lyse not only *E. cloacae* but also *Enterobacter ludwigii* and *Enterobacter kobei* (45). *E. cloacae* phage ECLFM1 demonstrated high phage infectivity, lysing 96 (78%) of tested strains (46). In our study, we isolated *Enterobacter* phages with varying host ranges, including both narrow and broad spectra. Notably, ФEBU8, a broad-spectrum phage, was capable of lysing multiple species, including *E. bugandensis*, *E. cloacae*, and *E. hormaechei*. For the first time, we report that *Enterobacter* phages can also lyse *A. baumannii*, although the lytic activity is relatively weak. This finding is consistent with a recent preprint publication on BioRxiv, which demonstrated that *Enterobacter* jumbo phages exhibit broad host ranges across pathogenic Gammaproteobacteria, including *Escherichia coli*, *Klebsiella aerogenes*, *Serratia marcescens*, *Salmonella* spp., *Shigella* spp., *Providencia* spp., *Citrobacter* spp., and *Cronobacter sakazakii* (47). Meanwhile, an *Enterococcus* phage ФEF24 was able to lyse *E. hormaechie*. Similarly, it is reported that a *Salmonella Enteritidis* bacteriophage, SS3e, also lysed E. cloacae stains (48). We know that host range is a central trait in understanding phage biology and determined by a range of molecular interactions between the phage and host during the infection cycle. Recent studies have shed light on the molecular mechanisms that enable phages to infect multiple hosts (49). Importantly, bacteriophage host range is not a fixed property; it can evolve over time and display unexpected plasticity (50). So far, the precise mechanism by which *Enterobacter* phages infect *A. baumannii* remains unclear. However, given the reported coinfection of *A. baumannii* and the *E. cloacae* complex in clinical settings (51), our phages hold promise for treating such polymicrobial infections.

In gram-negative bacteria, numerous surface-exposed components, such as outer membrane proteins (OMPs), lipopolysaccharide (LPS), exopolysaccharides, CPSs, flagella, and pili, can serve as phage receptors (52). OmpA, a porin with a two-domain structure, consists of an 8-stranded β-barrel pore in the outer membrane and flexible extracellular loops, making it a potential receptor for phages (53). OmpA has been identified as a receptor for T-even-like phages (38, 54) and the *E. coli* coliphage LHE83 (55). To the best of our knowledge, we are the first to demonstrate that OmpA serves as a receptor for *E. cloacae*. Notably, three spontaneous ФECL22-resistant mutants exhibited termination point mutations in their *ompA* genes, and reintroduction of the intact OmpA protein restored phage sensitivity to wild-type levels. However, some other ФECL22-resistant *Enterobacter* strains retained an intact OmpA protein; however, ФECL22 was unable to adsorb on them. This observation suggests the potential involvement of an auxiliary receptor in ФECL22-sensitive strains, which may be absent in ФECL22-resistant strains. This finding aligns with reports of *K. pneumoniae* phages NPat and BMac, which rely on CPS as the primary receptor and an outer membrane protein as the secondary receptor (56). In such cases, both receptors are essential for successful phage adsorption and DNA injection, illustrating how dual receptor systems influence phage-host interactions. This mechanism has profound implications for understanding the evolution of phage resistance and factors determining host range. Similarly, CPS plays a critical role in the virulence of ECC strains, and we hypothesize that different CPS subtypes dictate the host range of *Enterobacter* phages.

In our phage cocktail, the phages exhibited notable differences in genome sizes: ФEBU8 and ФECL22 had large genomes exceeding 170 kb, whereas ФECL30’s genome was smaller, around 40 kb. The majority of *Enterobacter* phages typically have large genomes. For example, the *E. cloacae* phages ECLFM1 and EBP have genomes of 172 kb and 179 kb, respectively (46, 57). However, smaller-genome *Enterobacter* phages also exist, such as MJ2 (40 kb) (16), PZJ0206 (40 kb) (58), and Ec_L1 (51 kb) (59). Accordingly, ФEBU8 and ФECL22 belong to the large-genome class of *E. cloacae* phages, whereas ФECL30 represents the small-genome group. Phylogenetic analysis placed these phages in different clades, suggesting distinct evolutionary backgrounds. On the other hand, most *E. cloacae* phages have burst sizes exceeding 100 PFU/cell (57, 58). In contrast, ФECL22 exhibited a low burst size of 19 PFU/cell, similar to other large-genome *E. cloacae* phages, which have burst sizes between 15 and 33 PFU/cell (60). Although the underlying reasons for burst size variation within the same bacterial species remain unclear, ФECL22 showed high efficiency in plating against *E. cloacae in vitro*. Therefore, our selection of three *E. cloacae* phages with distant phylogenetic relationships may help in counteracting phage-resistant bacteria. In clinical settings, phage cocktails designed to target multiple pathogens may offer a more comprehensive solution, but their effectiveness depends on careful selection of phages with minimal cross-reactivity or interference (61). Exploring these interactions in polymicrobial infection models is critical to optimizing phage therapy protocols. Although our current study did not address these interactions directly, our findings lay the groundwork for future research to investigate the efficacy of phage cocktails in polymicrobial infections. We envision that such studies could leverage sequencing and metagenomic analyses to identify key pathogens and develop tailored phage combinations for complex clinical scenarios.

Unlike conventional antimicrobial agents, phages can self-propagate during the bactericidal process. This means that as long as sensitive bacterial hosts are present, phage populations increase rapidly, and ultimately suppress bacterial growth. In a previous study, we observed that *A. baumannii* phage therapy showed dose-dependent efficacy in treating *A. baumannii* bacteremia (17). In this study, although the final survival rates did not significantly differ among phage treatment groups of varying doses, clinical symptoms and body weight outcomes indicated that the middle dose (1 × 10⁶ PFU/mouse) outperformed the other two groups. This group had the highest phage titers and the lowest bacterial loads in organs, as well as the smallest loss in body weight. It has been reported that phage dosage can influence the generation of neutralizing antibodies, which may inactivate phages at higher doses (62). However, the short timeframe of our study likely precluded the development of a significant humoral immune response. Phage-neutralizing antibody was detected in serum from 10 to 42 days post-treatment (63). Additionally, innate immune responses to high phage doses may also play a role, potentially influencing bacterial clearance or triggering inflammatory side effects. Understanding how phage dosage interacts with the immune system, both innate and adaptive, remains a critical area for future investigation. These interactions must be carefully balanced to optimize therapeutic outcomes while minimizing the risk of adverse immune-mediated effects.

Bacteremia is a severe condition where immediate treatment can be lifesaving. Our study highlights the importance of timely intervention with phage therapy, showing that earlier administration leads to better outcomes. Administering phages immediately after infection saved 100% of the infected mice, although delaying treatment to 6 h POI reduced survival to 50%, with these survivors experiencing significant weight loss. Prophylactic phage administration 24 h before infection did not prevent mortality, likely because no phage remained detectable 24 h post-administration, consistent with findings that *A. baumannii* phage ФAb4B disappears from the bloodstream within 12 h (17). However, a combined regimen of prophylactic phage administration at −24 h with delayed treatment at 6 h (“−24 + 6”) achieved 100% survival. This approach also resulted in lower bacterial loads in the blood, higher phage titers in the spleen, elevated white blood cell counts, and reduced cytokine levels compared with the “6” regimen alone. It remains unclear whether the increased white blood cell count directly contributed to bacterial clearance or was a result of effective bacterial reduction, warranting further investigation into the underlying mechanism.

Phage therapy can serve as either a preventive measure or a therapeutic approach. Prophylactic administration of bacteriophage cocktails has shown promise in multiple studies. For instance, a cocktail termed FOP (foodborne outbreak pill) administered prophylactically for 2 days, once daily, delayed the onset of *Salmonella* infection in OMM12 mice (64). Similarly, preventive administration of siphovirus PSE over three days, once every 24 h, reduced *S. Enteritidis* colonization more effectively than post-challenge treatment (65). In both cases, the interval between the last phage administration and bacterial challenge exceeded 24 h; however, prophylactic phage therapy remained effective. Further supporting this, a single intraperitoneal injection of a bacteriophage cocktail targeting *A. baumannii*, *K. pneumoniae*, *P. aeruginosa*, and *S. aureus* provided 100% survival in mice challenged with *K. pneumoniae* B2580 strain 24 h post-administration (66). Although the study did not report whether residual phages were detectable after 24 h, it highlights the potential for prophylactic applications. In another example, oral administration of a phage cocktail up to 24 h before a *V. cholerae* challenge prevented intestinal colonization and cholera-like diarrhea in mouse and rabbit models (67). Measurement of phage titers confirmed that the ICP phages remained viable in the intestinal tract for at least 24 h in the absence of their host. These findings collectively suggest that the efficacy of prophylactic phage administration depends on the persistence of phages within the host. *In vivo*, phages are typically cleared in the absence of their bacterial hosts, emphasizing the need for optimized dosing strategies to maximize their preventive potential. Future research should focus on understanding the factors that influence phage persistence and efficacy to expand the therapeutic utility of phages in both prophylactic and therapeutic settings.

In conclusion, we isolated and characterized novel lytic phages targeting *Enterobacter* species, and our phage cocktail demonstrated efficacy in protecting mice from *E. cloacae*-induced bacteremia. The cocktail’s protective efficiency was dose-dependent, and prophylactic use provided a valuable window for delayed treatment, offering a promising strategy for combating severe bacterial infections.

## SUPPLEMENTAL MATERIAL

Supplemental figures aac.01683-24-s0001.pdf Figures S1 to S7.

Table S1 aac.01683-24-s0002.docx Bacterial strains, plasmids, and primers used in this study.

Table S2 aac.01683-24-s0003.xlsx Annotation of three phages.

Table S3 aac.01683-24-s0004.xlsx SNP of phage-resistant mutants.

ASM does not own the copyrights to Supplemental Material that may be linked to, or accessed through, an article. The authors have granted ASM a non-exclusive, world-wide license to publish the Supplemental Material files. Please contact the corresponding author directly for reuse.
