# Supplementary material for: Optimizing phage therapy for carbapenem-resistant Enterobacter cloacae bacteremia: insights into dose and timing
Source: Antimicrob Agents Chemother. 2025 Feb 26;69(4):e01683-24. doi: 10.1128/aac.01683-24 (PMC11963603; doi:10.1128/aac.01683-24)
Supplement: Supplemental figures — Figures S1 to S7. [file aac.01683-24-s0001.pdf]

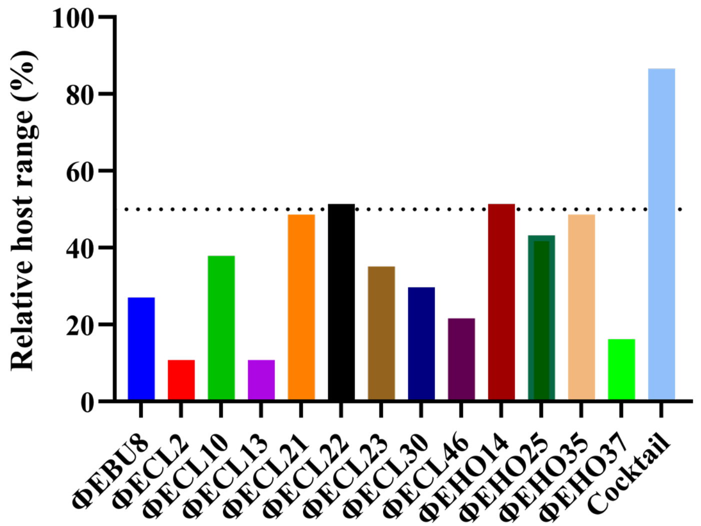

Figure S1. Host range of isolated *Enterobacter* phages. The relative recognition rates of the isolated Enterobacter phages were evaluated against 37 ECC strains. Each phage's ability to lyse specific strains is presented, highlighting their spectrum of activity across the panel.

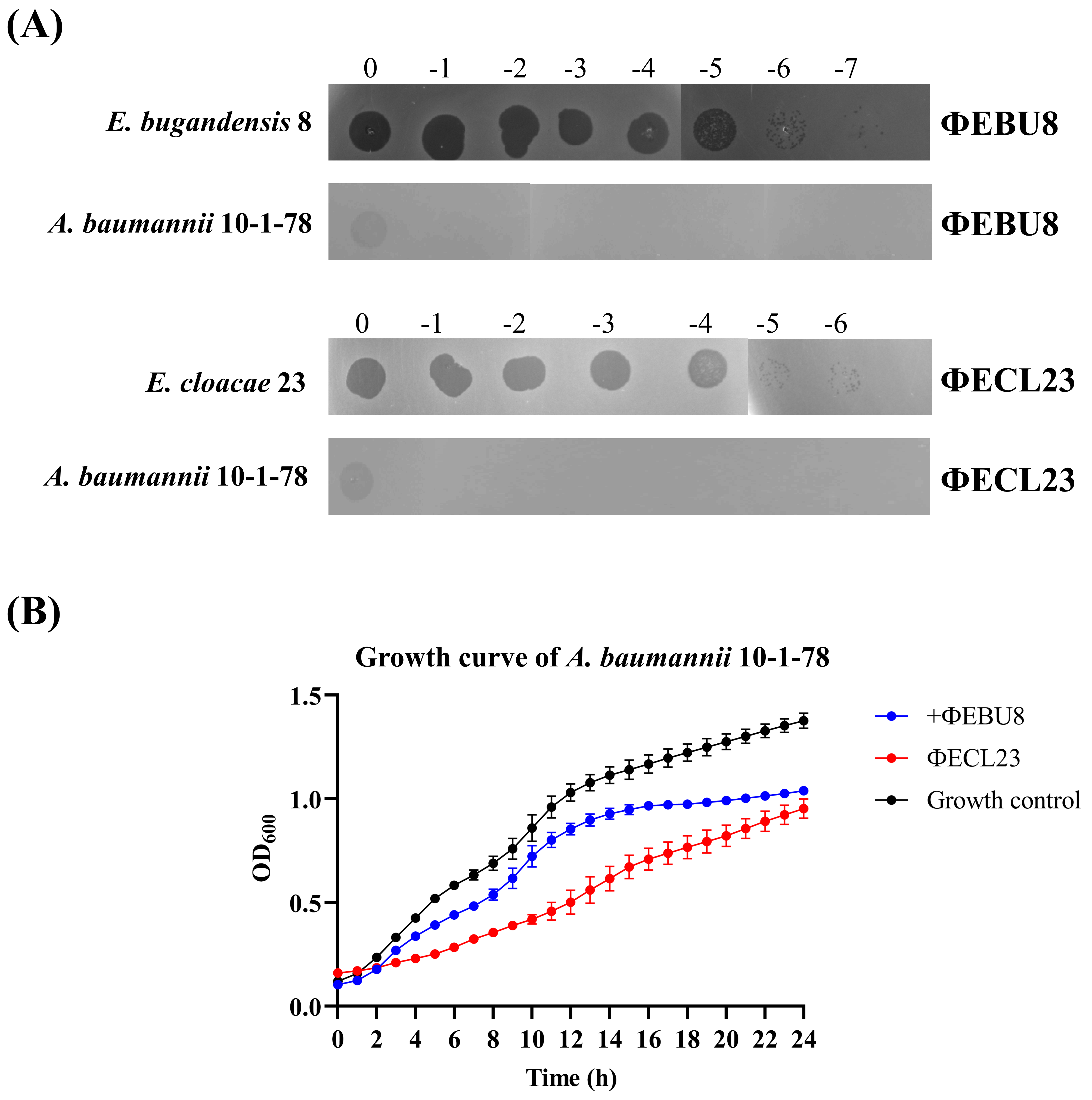

Figure S2. Confirmation of *Enterobacter* phage lysis of *Acinetobacter baumannii* Strain. (A) Efficiency of plating (EOP) of phages ΦEBU8 and ΦECL23 against their original bacterial hosts and *A. baumannii* 10-1-78. (B) Growth curve of *A. baumannii* 10-1-78 in the presence of ΦEBU8 or ΦECL23. Bacterial cultures without phage treatment served as the growth control.

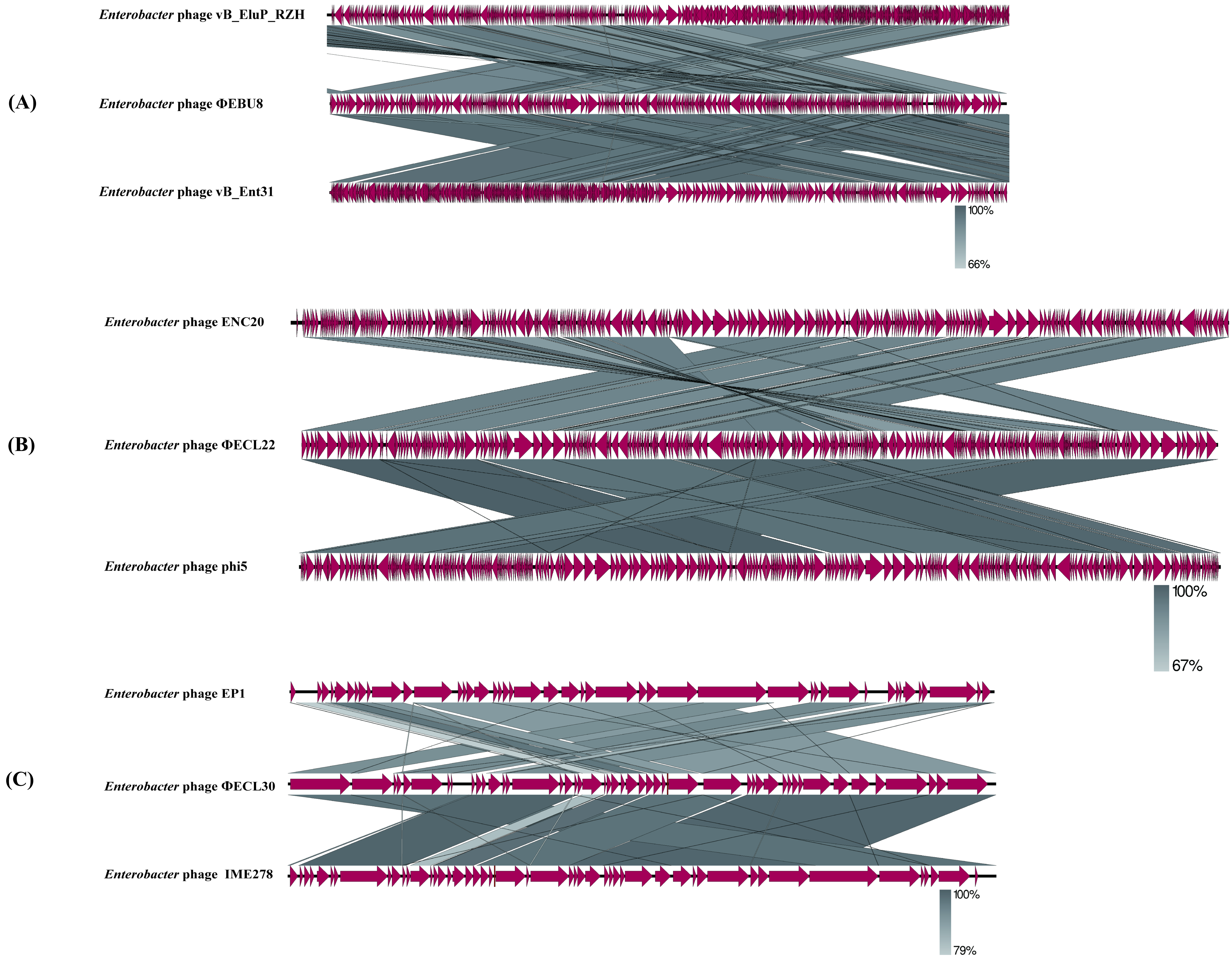

Figure S3. Comparative genome analysis of three *Enterobacter* phages alongside their respective homologous phages. Linear alignment of genomic sequences was conducted using Easyfig.

Capsid protein

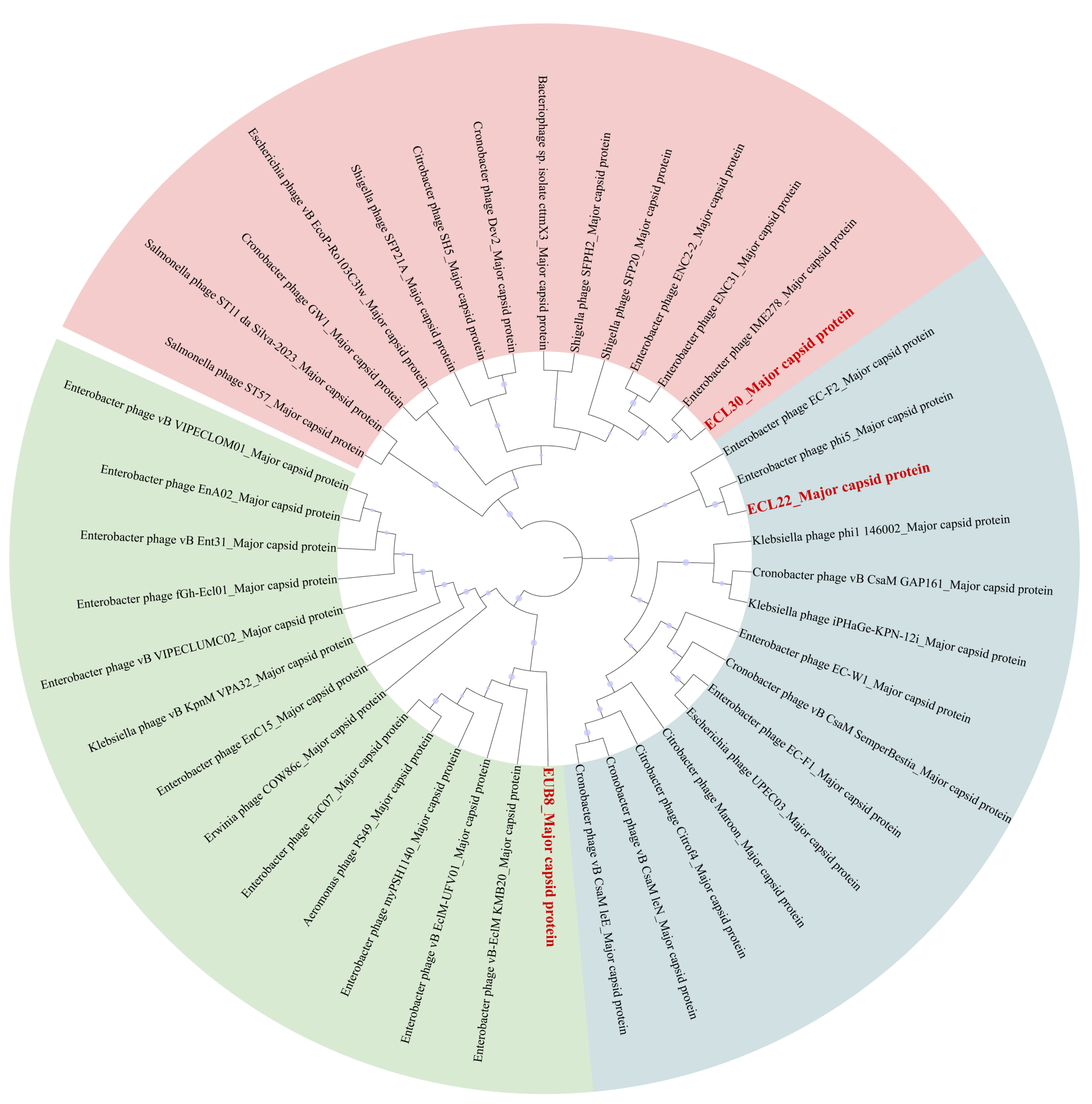

Terminase large subunit

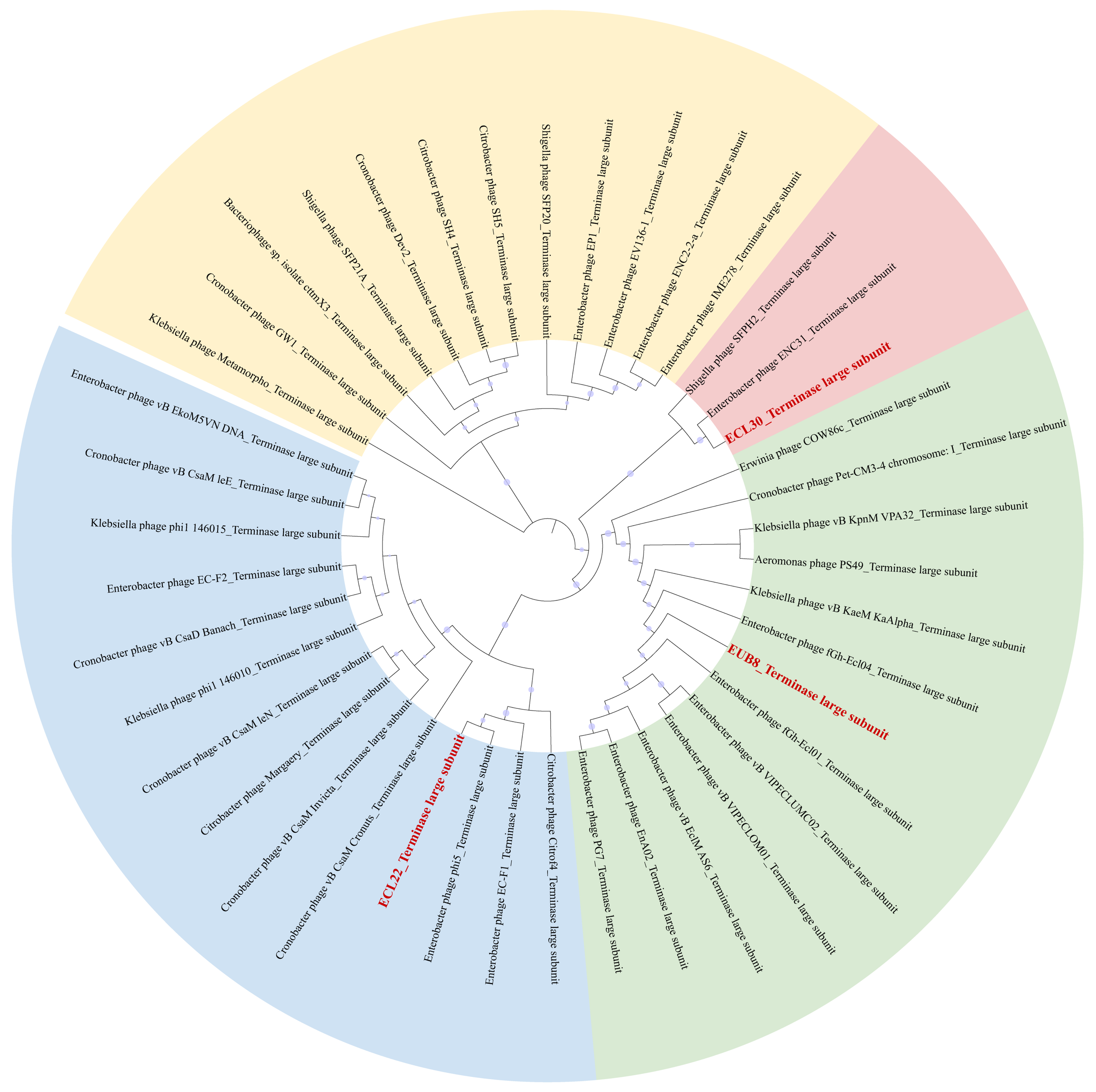

Figure S4. Phylogenetic trees based on the major capsid protein sequence (left) and the terminase large subunit protein sequence (right). Evolutionary relationships were analyzed using the Neighbor-Joining method in MEGA7. The bootstrap consensus trees, inferred from 500 replicates, represent the evolutionary history of the analyzed taxa. The phylogenetic tree was presented by Interactive Tree Of Life (ITOL).

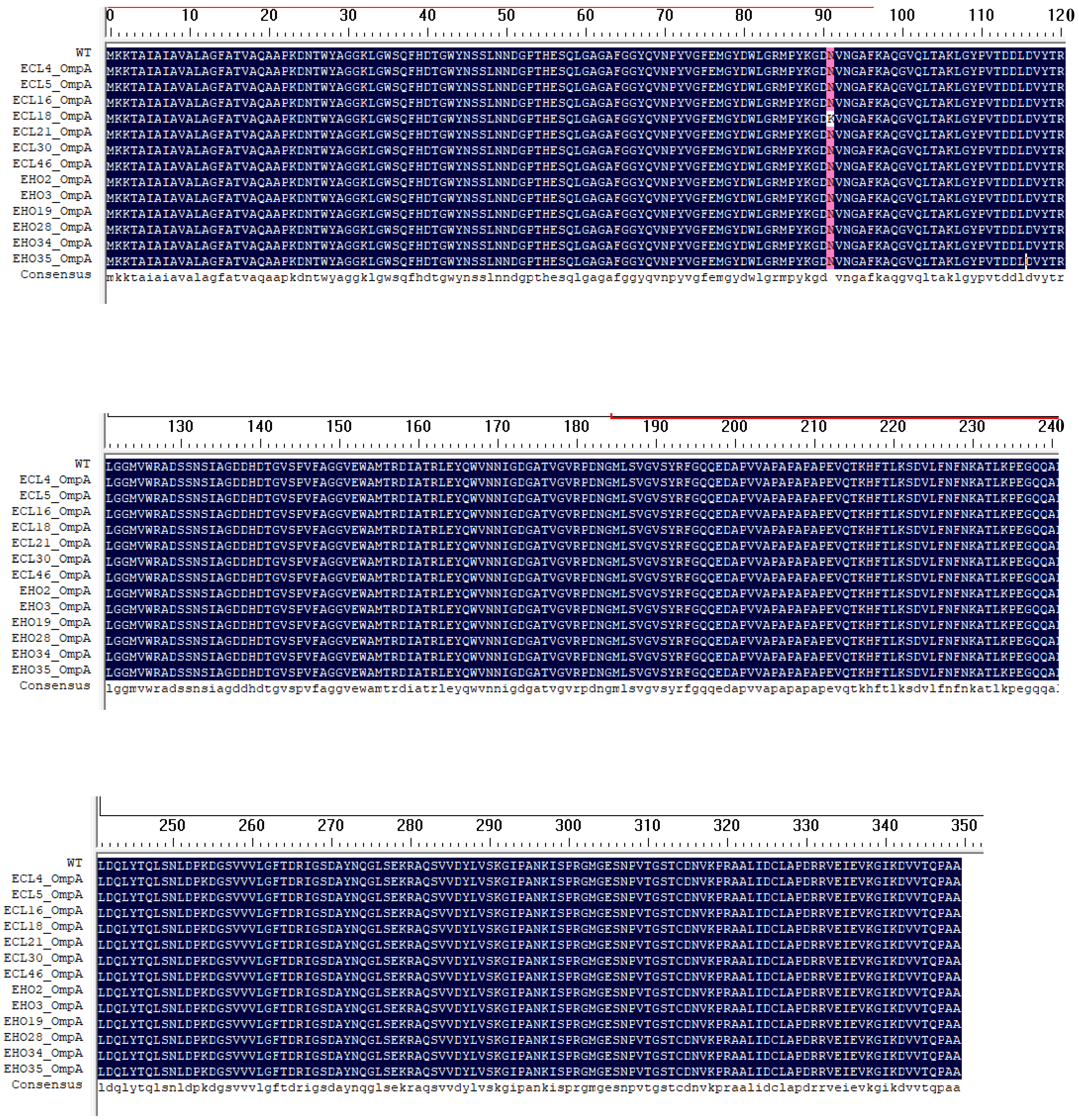

Figure S5. Multiple sequence alignment of OmpA protein of *E. cloacae* complex strains using DNAman software.

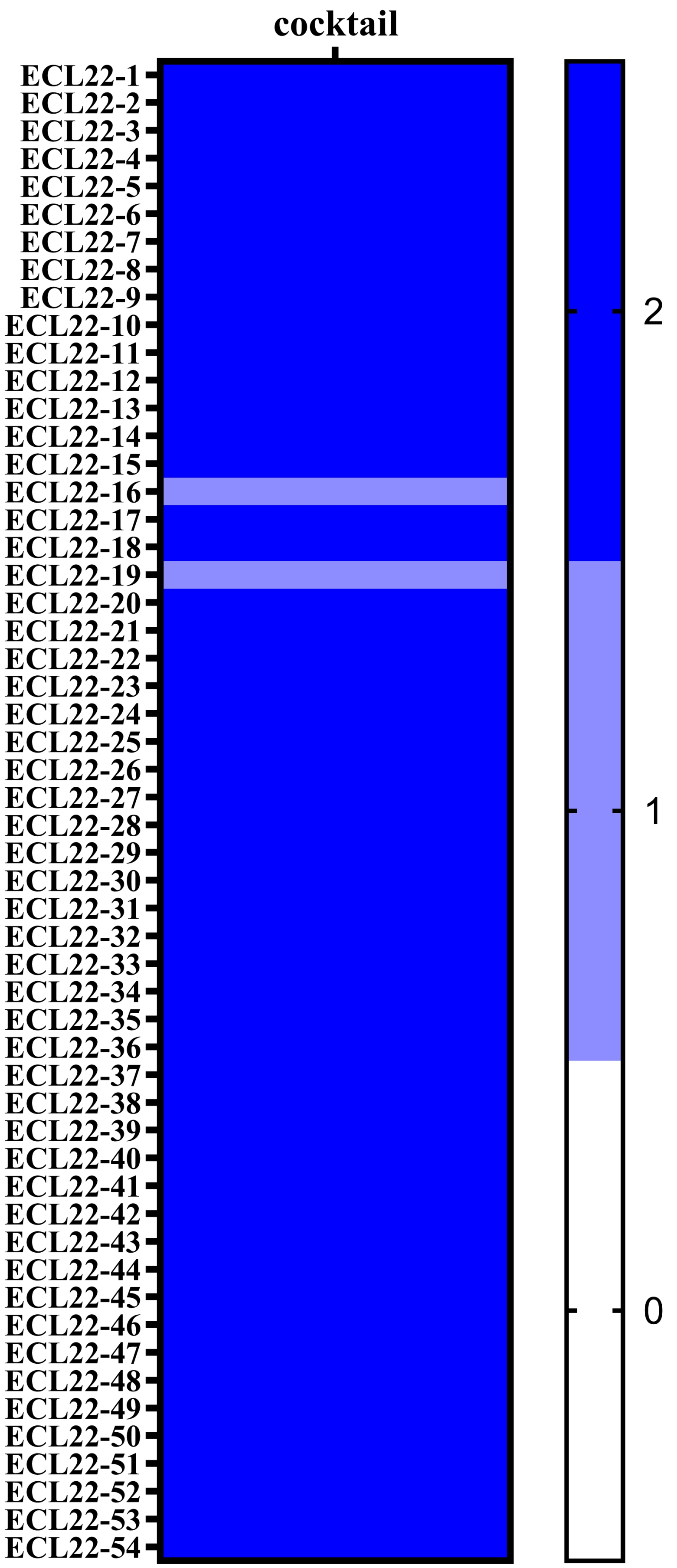

Figure S6. Phage sensitivity of bacterial strains isolated from liver, spleen and blood samples of infected mice treated with different doses of phage cocktail. Dark blue, large and clear plaque; light blue, vague plaque; white, no plaque.

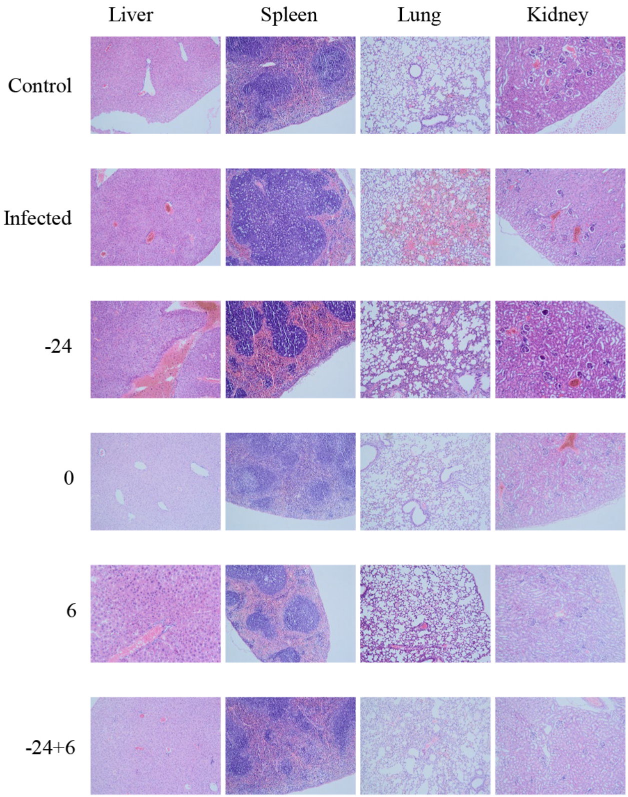

Figure S7. HE staining of liver, spleen, lungs, and kidneys of mice treated with phage cocktail at different time intervals. Samples were taken at 24 h POI.
