# Supplementary material for: Optimizing phage therapy for carbapenem-resistant Enterobacter cloacae bacteremia: insights into dose and timing
Source: Antimicrob Agents Chemother. 2025 Feb 26;69(4):e01683-24. doi: 10.1128/aac.01683-24 (PMC11963603; doi:10.1128/aac.01683-24)
Supplement: Table S1 — Bacterial strains, plasmids, and primers used in this study. [file aac.01683-24-s0002.docx]

**Table S1. Bacterial strains, plasmids and primers used in this study.**

| **Bacteria** | **Antimicrobial resistance** | **Sources or references** |  |
| --- | --- | --- | --- |
| *E. bugandensis 8* | Carbapenemase-resistant | sputum |  |
| *E. cloacae 1* | Carbapenemase-sensitive | sputum |  |
| *E. cloacae 4* | Carbapenemase-resistant | sputum |  |
| *E. cloacae 5* | Carbapenemase-sensitive | sputum |  |
| *E. cloacae 6* | Carbapenemase-resistant | sputum |  |
| *E. cloacae 10* | Carbapenemase-sensitive | sputum |  |
| *E. cloacae 12* | Carbapenemase-sensitive | urine |  |
| *E. cloacae 13* | Carbapenemase-sensitive | blood |  |
| *E.cloacae 13-3* | Carbapenemase-resistant | ascites |  |
| *E.cloacae 16* | Carbapenemase-resistant | sputum |  |
| *E.cloacae 18* | Carbapenemase-sensitive | sputum |  |
| *E. cloacae 20* | Carbapenemase-resistant | cerebrospinal fluid |  |
| *E. cloacae 21* | Carbapenemase-resistant | sputum |  |
| *E. cloacae 22* | Carbapenemase-resistant | cerebrospinal fluid |  |
| *E. cloacae 23* | Carbapenemase-resistant | sputum |  |
| *E. cloacae 26* | Carbapenemase-resistant | blood |  |
| *E. cloacae 27* | Carbapenemase-resistant | sputum |  |
| *E. cloacae 30* | Carbapenemase-sensitive | sputum |  |
| *E. cloacae 32* | Carbapenemase-sensitive | sputum |  |
| *E. cloacae 46* | Carbapenemase-sensitive | urine |  |
| *E. hormaechei 2* | Carbapenemase-sensitive | sputum |  |
| *E. hormaechei 3* | Carbapenemase-resistant | sputum |  |
| *E. hormaechei 7* | Carbapenemase-sensitive | sputum |  |
| *E. hormaechei 9* | Carbapenemase-sensitive | sputum |  |
| *E. hormaechei 11* | Carbapenemase-sensitive | sputum |  |
| *E. hormaechei 14* | Carbapenemase-sensitive | rests |  |
| *E. hormaechei 15* | Carbapenemase-sensitive | sputum |  |
| *E. hormaechei 17* | Carbapenemase-resistant | fester |  |
| *E. hormaechei 19* | Carbapenemase-resistant | wound secretion |  |
| *E. hormaechei 25* | Carbapenemase-resistant | sputum |  |
| *E. hormaechei 28* | Carbapenemase-resistant | fester |  |
| *E. hormaechei 31* | Carbapenemase-sensitive | sputum |  |
| *E. hormaechei 33* | Carbapenemase-sensitive | catheter |  |
| *E. hormaechei 34* | Carbapenemase-sensitive | fester |  |
| *E. hormaechei 35* | Carbapenemase-sensitive | sputum |  |
| *E.hormaechei 37* | Carbapenemase-resistant | sputum |  |
| *E.hormaechei 47* | Carbapenemase-sensitive | lavage fluid |  |
| *E. coli* Stellar | Apramycin-sensitive | TaKaRa Bio |  |
| *A. baumannii 48* | Carbapenemase-sensitive | sputum |  |
| *E. faecalis24* | / | urine |  |
| *A. baumannii10-1-15* | Carbapenemase-resistant | lavage fluid |  |
| *A. baumannii10-1-23* | Carbapenemase-resistant | sputum |  |
| *A. baumannii10-1-71* | / | sputum |  |
| *A. baumannii10-1-78* | / | sputum |  |
| *A. baumannii10-1-79* | / | sputum |  |
| *A. baumannii YQ4* | pandrug resistant, apramycin-sensitive | [1] |  |
| *S. aereus 9-4MK* | / | hydrothorax and ascite |  |
| *S. aereus 9-11MK* | / | sputum |  |
| *S. aereus 9-46 MK* | / | sputum |  |
| *E. coli 13-1MK* | Carbapenemase-resistant | sputum |  |
| *E. coli 13-2MK* | Carbapenemase-resistant | sputum |  |
| *P. aeruginosa 1-22MK* | Carbapenemase-sensitive | sputum |  |
| *P. aeruginosa 1-41MK* | Carbapenemase-resistant | sputum |  |
| *K. pneumoniae 5-1-4MK* | Carbapenemase-resistant | sputum |  |
| *K. pneumoniae 5-1-8MK* | Carbapenemase-resistant | whole blood |  |
| *K. pneumoniae 5-1-47MK* | Carbapenemase-resistant | sputum |  |
| *K. pneumoniae 84* | pandrug resistant, apramycin-sensitive | Laboratory preservation |  |
| *E. cloacae* R3 | Carbapenemase-resistant | phage-resistant mutant from E. cloacae 22 |  |
| *E. cloacae* R4 | Carbapenemase-resistant | phage-resistant mutant from E. cloacae 22 |  |
| *E. cloacae* R6 | Carbapenemase-resistant | phage-resistant mutant from E. cloacae 22 |  |
|  |  |  |  |
| **Plasmids** | **Antimicrobial resistance** | **Use** | **References or sources** |
| pBECAb-Apr | Apramycin-resistant | base editing plasmid in *A. baumannii* | [2] |
| pHERD20T | Carbenecillin-resistant | expression plasmid with pBAD promoter | [3] |
| pWCab24T | Apramycin-resistant | expression plasmid with pBAD promoter | this study |
| pWCab24T-OmpAECL | Apramycin-resistant | to express OmpA of *E. cloacae* | this study |
| pWCab24T-OmpAECO | Apramycin-resistant | to express OmpA of *E. coli* | this study |
|  |  |  |  |
| **Primers** | **Sequences** | **Use** |  |
| 16s-27F | AGAGTTTGATCCTGGCTCAG | bacterial identification |  |
| 16s-1492R | GGTTACCTTGTTACGACTT |  |  |
| BECAb-qR | GTGATATGTCCTCCTCTCTTC | to linearize pBECAb-Apr |  |
| BECAb-bF | GATATCGAATTCCTGCAGC |  |  |
| araC-F | ATAAAGTTGCAAGCT ATGCAGCGGAAAGTATACC | to amplify the araC-pBAD fragment from pHERD20T |  |
| araC-R | GCTCTAGAACTAGTG GTAAAACGACGGCCAGTG |  |  |
| ompA-F | GATCTGAT AAG AAT TCG ATGAAAAAGACAGCTATCGCGA |  |  |
| ompA-Ecl-R2 | GGCCAGTGCCAAGCT TTATGCCGCAGGCTGAGTTAC | to amplify ompA gene from *E. cloacae* |  |
| ompA-Eco-R2 | GGCCAGTGCCAAGCT TTAAGCCTGCGGCTGAGTTAC | to amplify *ompA* gene from *E. coli* |  |
| SCN-ECLompA-F | CGGACTTCACACTTGTAAG | To screen the *ompA* gene in other ECL strains |  |
| SCN-ECLompA-R | ACAAAGAGAAGTATGCCAG |  |  |
| phage Ecl8-qR | CCCTCCAAGTTCAGATTGG | To determine the genome structure of phage EBU8 |  |
| phage Ecl8-bF | CCCTCCAAGTTCAGATTGG |  |  |
| phage Ecl22-qR | gactgatgcggaaaatactgta | To determine the genome structure of phage ECL22 |  |
| phage Ecl22-bF | acgttacgcgggttatcttcac |  |  |
| phage Ecl30-qR | ACGTAGAGCGGTCAGCTC | To determine the genome structure of phage ECL30 |  |
| phage Ecl30-bF | TGATGCTGGATGCATACCG |  |  |

1. Wang, W.X., et al., *Phage therapy combats pan drug-resistant Acinetobacter baumannii infection safely and efficiently.* Int J Antimicrob Agents, 2024. **64**(2): p. 107220.

2. Wang, Y., et al., *A Highly Efficient CRISPR-Cas9-Based Genome Engineering Platform in Acinetobacter baumannii to Understand the H(2)O(2)-Sensing Mechanism of OxyR.* Cell Chem Biol, 2019. **26**(12): p. 1732-1742.e5.

3. Qiu, D., et al., *PBAD-based shuttle vectors for functional analysis of toxic and highly regulated genes in Pseudomonas and Burkholderia spp. and other bacteria.* Appl Environ Microbiol, 2008. **74**(23): p. 7422-6.
